# The Effect of Farming Systems and Cultivars on the Qualitative and Quantitative Composition of Bioactive Compounds in Winter Wheat (*Triticum aestivum* L.)

**DOI:** 10.3390/molecules30040902

**Published:** 2025-02-15

**Authors:** Iwona Kowalska, Agata Soluch, Jarosław Mołdoch, Krzysztof Jończyk

**Affiliations:** 1 Department of Phytochemistry, Institute of Soil Science and Plant Cultivation-State Research Institute, Czartoryskich Str. 8, 24-100 Pulawy, Poland; asoluch@iung.pulawy.pl (A.S.); jmoldoch@iung.pulawy.pl (J.M.); 2 Department of Agroecology and Economics, Institute of Soil Science and Plant Cultivation-State Research Institute, Czartoryskich Str. 8, 24-100 Pulawy, Poland; kjonczyk@iung.pulawy.pl

**Keywords:** winter wheat, phenolic acids, UPLC-DAD-MS, alkylresorcinols, UPLC-PDA-MS/MS, TLC-DPPH^•^, bioactive compounds, monoculture system

## Abstract

*Triticum aestivum* L. subsp. *spelta* (cv. Rokosz) and common winter wheat *Triticum aestivum* L. subsp. *aestivum* (cv. Arktis, Belissa, Estivus, Fidelius, Hondia, Jantarka, KWS Ozon, Linus, Markiza, Ostka Strzelecka, Pokusa) grown in an organic farming system were analyzed and compared. Furthermore, the productivity of four common wheat cultivars (cv. Fidelius, Hondia, Jantarka, KWS Ozon) grown under four different (organic, conventional integrated, and monoculture) farming systems was compared. Using UPLC-DAD-MS, UPLC-PDA-MS/MS, and TLC-DPPH^•^, nine phenolic acids, nine alkylresorcinols, and their antiradical activity were identified and quantified. In the organic farming system, the highest yield was observed for *T. aestivum* L. subsp. *aestivum* cv. Fidelius (4.17 t/ha). Infections of wheat cultivars were low or at a medium level. The highest resistance to *Fusarium* fungi was shown by cv. Fidelius, which also exhibited the highest alkylresorcinol content and antioxidant capacity. The total phenolic acid content was highest in cv. Rokosz (1302.3 µg/g), followed by common winter wheat cultivars cv. Linus (1135.1 µg/g) and cv. Markiza (1089.6 µg/g). The potential of winter wheat cultivars for human health and their suitability for cultivation in different production systems was determined, showing significant differences in bioactive compounds depending on cultivars, systems, and years.

## 1. Introduction

The primary cereal cultivated in Poland is common wheat, *Triticum aestivum* L. ssp. *aestivum*, also known as soft wheat. This plant grows on 221 million hectares worldwide, and global production, for example, in 2021 amounted to 771 million tonnes [1]. Wheat is the most important cereal both in Poland and throughout the world. This is due to the versatility of grain use and the large production potential of this plant. It is the basic raw material in the milling and baking industries. The production of wheat grain with good technological parameters requires the use of innovative technologies and biological progress. Wheat grain, as a raw material for consumption and feed, is of strategic importance as it determines food security [2].

Currently, especially in highly developed countries, wheat is mostly grown intensively in the conventional system (CON), which relies on the extensive use of mineral fertilizers and chemical pesticides [3]. The opposite of this system is organic farming (ORG), which aims to promote ecosystem health by reducing external inputs, such as synthetic fertilizers, synthetic pesticides, and genetically modified organisms. The production constraints associated with organic farming are often greater than those that conventional production faces. Increased weed pressure and soil nutrient deficiencies, particularly in nitrogen and phosphorus, are more common in this system, which may lead to crop yield reductions. To minimize these constraints and comply with the requirements for organic certification products, growers use agricultural practices such as crop rotation, varying sowing dates and rates, intercropping, and the use of animal and green manures, as well as cultivar selection [4].

Established in 2001, the European Initiative for Sustainable Development of Agriculture (EISA) promotes and defends coherent principles of integrated production (INT) in the European Union. In Poland, since 14 June 2007, due to the decision of the Minister of Agriculture and Rural Development, integrated production, as understood based on Art. 5, par. 1 of the Act on Plant Protection, has been recognized as a national food quality system [5]. Integrated farming is a management system that aims to deliver more sustainable agriculture without compromising the quality or quantity of agricultural products. The INT system combines modern tools and technologies with traditional practices according to specific sites and situations, often employing many different cultivation techniques [6].

In conclusion, the most widespread and well-known farming systems (CON, ORG, and INT) tend to minimize expenses incurred for farming tillage and use specialized crop rotations with 2–3 plant species that require the same or similar cultivation techniques [7,8]. The cultivation of cereals in a monoculture system (MONO) is guided by completely different assumptions. Winter wheat in monoculture is occasionally recommended as the most financially competitive cultivation system. Unfortunately, compared with wheat grown after oilseed rape, peas, or oats, the yield of wheat after wheat is reduced by 8–57%, depending on the site, weather conditions, and crop management [9].

Phenolic compounds in the grains of these cereals have become the focus of research due to their antioxidative activities. These properties may be a significant factor in protecting humans against the development of some chronic diseases (such as heart disease, cancer, and atherosclerosis) because of the scale of cereal consumption [10,11]. Wheat-based food ingredients, rich in natural bioactive compounds, can serve as an ideal basis for the development of functional foods designed to improve the health of millions of consumers [12]. Very important is the fact that the content of phenols in cereal grains depends on the genotype, environmental conditions, agrotechnical factors, and their interactions. These factors are also known to affect the growth, development, yield, and quality parameters of grain, including the content of the bioactive compounds discussed [13]. The health-promoting phytonutrients of wheat grains include, among others, alkylresorcinols (ARs) and phenolic acids (PAs), and the presence of these secondary metabolites in the daily diet can potentially increase resistance to the previously mentioned lifestyle diseases [10]. Referring to the whole plant, of course, it is not only in the grains that the compounds mentioned occur. There are many sources in the literature confirming the presence of phenols in other organs of the wheat [14,15,16,17,18].

It is common knowledge that the level of phenolic compounds, including ARs and Pas in cereals, depends on the cultivar and growth conditions [19]; therefore, we conducted studies on these relationships in four different cultivar systems. It should be emphasized that our research covers four farming systems: organic (ORG), integrated (INT), conventional (CON), and monoculture (MONO), while most of the studies conducted so far have been limited to comparing only the first three cultivation methodologies. The analysis of the literature shows that these four cultivation systems were compared for the first time, including those not taken into account so far, including the monoculture system. Therefore, the aim of this study was to demonstrate the effect of the four cultivation systems, CON, ORG, INT, and MONO, on the antioxidant activity and content of selected phenolic compounds in the grain of four winter wheat cultivars. The knowledge gathered from the conducted research can help direct the breeding or engineering of these plants to obtain higher concentrations of these compounds while maintaining higher-yielding cultivars.

## 2. Results and Discussion

### 2.1. Results of the Organic Farming System

#### 2.1.1. Biometric Analyses, Grain Yield, Evaluation of Plant Infestation Using Pathogen and *Fusarium* spp. Occurrence

In 2017, the evaluated cultivars yielded in the range of 1.8 to 4.17 t/ha (Table S1). A significant impact on wheat yield was due to unfavorable weather patterns and an increased occurrence of brown rust (Table S2). Excessive soil moisture in October (100 mm of rainfall exceeding twice the long-term norm) made it impossible to carry out effective care treatments, contributing to increased weed infestation (66 pcs·m^−2^). In addition, in January, there were low temperatures, which due to the lack of snow cover, caused damage to plants and thinning of the field.

In 2018, wheat yielded at a similar level to that of 2017, obtaining, depending on the cultivar, a grain yield in the range of 2.78–3.97 t/ha. A significant factor limiting productivity in 2018 was the lack of rainfall during the grain formation period, which directly affected grain yield.

Among the evaluated cultivars, regardless of the year, the group of cultivars with above-average yield included Bellisa, Estivus, Fidelius, and Jantarka (Table S1). The high productivity of the above-mentioned cultivars is likely associated with their ability to produce grain with greater ripeness and, in 2017, a higher number of ears. The spelt winter wheat cultivar (cv. Rokosz) yielded below average, depending on the year, with yields 4 to 45% lower than average.

Infections of winter wheat cultivars during the studied period were low or at a medium level. For most diseases, no significant differences were found between the cultivars. The relatively wet and cool conditions in May and June, along with large temperature fluctuations in 2017, could have had an impact on the infection of wheat by *Pucinia recondita* and *Pucinia striiformis* (Table S2). The infestation of winter wheat cultivars by *Pucinia recondita* in 2017 ranged from 3.8% for cv. Belissa to 51.5% for cv. Ostka Strzelecka and 54.2% for cv. Arktis. In the same year, greater diversity of disease symptoms among cultivars was also found in the case of *Pucinia striiformis.* The group of cultivars with the highest infestation by this pathogen included the following cultivars: Ostka Strzelecka—11.1%, Pokusa—12.7%, and Belissa—18.0%. A smaller leaf infestation of winter wheat by *Puccinia striiformis* was observed in the following cultivars: Jantarka—0.1%, KWS Ozon—0.2%, Linus—0.3%, Estivus—1.3%, and spelt wheat Rokosz—1.7%. In the case of *Septoria* spp. and *Drechslera tritici repentis*, the infestation of cultivars was low, and the differences in leaf damage were mostly statistically insignificant (Table S2).

#### 2.1.2. Chemical Analysis of Phenolic Acids (PAs)

**Figure 1:**
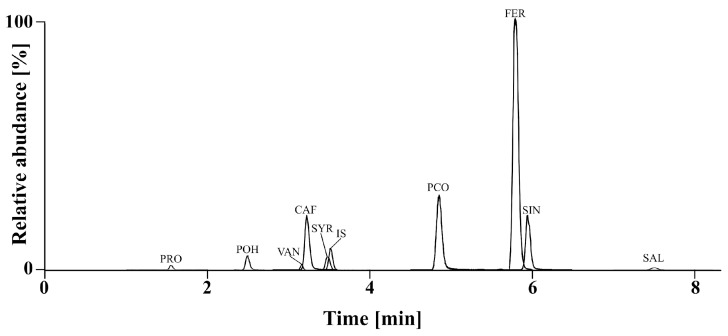
UPLC chromatograms in multiple reaction monitoring (MRM) mode of cv. Linus (2017) sample. Peak annotations: PRO—protocatechuic acid, POH—*p*-OH-benzoic acid, VAN—vanillic acid, CAF—caffeic acid, SYR—syringic acid, IS—Internal Standard (*m*-OH-Benzoic acid), PCO—*p*-coumaric acid, FER—ferulic acid, SIN—sinapic acid, SAL—salicylic acid.

Using an ultra-high-performance liquid chromatography method, nine phenolic acids (protocatechuic, *p*-OH-benzoic, vanillic, caffeic, syringic, *p*-coumaric, ferulic, sinapic, and salicylic acids) were identified and analyzed in all winter wheat cultivars (Figure 1).

The total PA content was highest in cv. Rokosz (mean 1302.3 µg/g), followed by cv. Linus (mean 1135.1 µg/g) and cv. Markiza (mean 1089.6 µg/g). These cultivars also showed the highest antioxidant activity. The lowest total content of PAs was shown for cv. Pokusa (mean 874.2 µg/g) and cv. Fidelius (mean 879.2 µg/g). In comparison, these values were higher than those for spring wheat cultivars grown under the same conditions and in the same years. The average PA content was 690.6 μg/g (in 2017) and 919.01 μg/g (in 2018) [10]. Ferulic acid was found to be the main phenolic acid in all samples. The other phenolic acids were found in much lower concentrations and can be ranked as follows: FER > PCO > SIN > CAF > VAN > SYR > PRO > POH > SAL. Ferulic acid constituted 64.5–83.8% (in 2017) and 70.6–84.9% (in 2018) of total phenolic acid content, while *p*-coumaric acid accounted for 2.6–18.1% (in 2017) and 2.9–18.1% (in 2018) (Table S3). The average FER content of the 12 cultivars was 827.6 µg/g, while Li et al. [20] showed that the range of FER content for 130 wheat genotypes ranged from 326.0 to 1171.0 µg/g, with a mean content of 664.0 µg/g. Kurasiak-Popowska et al. [21] observed significant differences in FER content among 100 cultivars and lines of winter wheat. Its mean concentration was 975 µg/g, and it accounted for 92.4% of all phenolic acids. Lower contents were found by Moore et al. [22] in American winter wheat cultivars and by Tian et al. [23] in four wheat genotypes.

The results of the analysis of variance (ANOVA) are summarized in Table S4. The year × cultivar interaction was significant for all phenolic acids and their activity. The year effect was significant for protocatechuic, *p*-OH-benzoic, vanillic, caffeic, syringic, *p*-coumaric, sinapic, and salicylic acid. No significant variation in ferulic acid levels, total phenolic acid content, and activity was detected, depending on the year of the study; however, winter wheat cultivars grown in 2017 had a higher total PA content (mean 1030 μg/g of grain) compared to 2018 (mean 1013 μg/g) (Table 1). The cultivar was significant for all phenolic acids and their activity. The variability coefficient was low and ranged from 1.99% (for salicylic acid) to 9.12% (for caffeic acid).

A differential effect of crop year, including weather conditions, on the content of particular phenolic acids was observed. The content of some acids (PRO, VAN, CAF, and PCO) was higher in 2017, while the content of others (POH, SYR, SIN, and SAL) was higher in 2018. Total phenolic acid content was highest in cv. Rokosz, both in 2017 and 2018 (mean 1302 µg/g), followed by cv. Linus (mean 1135 µg/g) and cv. Markiza (mean 1090 µg/g). These cultivars also showed the highest antioxidant activity. The lowest total content of PAs was shown for cv. Pokusa (mean 874 µg/g) and cv. Fidelius (mean 879 µg/g). The highest content of FRE was found in cv. Linus; PRO, POH, VAN, PCO, and SAL were highest in cv. Rokosz; and SYR was highest in cv. Markiza. Our results were consistent with previous studies that showed that weather conditions led to increased concentrations of phenolic compounds [24,25]. Several studies, however, have shown that the content of phenolic compounds in wheat significantly decreased during the maturation period [26,27,28]. We observed that ferulic acid, total phenolic acid, and activity were less sensitive to year variability (Table 1).

Statistically significant differences (*p* < 0.05) in the antiradical activity of individual cultivars were found. The antiradical activity of winter wheat grain is a result of the presence of ferulic, *p*-coumaric, sinapic, and caffeic acids (Table 1). In earlier research by Kowalska et al. [29], the mean antiradical activity of winter wheat cultivars grown in Poland was lower (0.177). Ferulic acid can prevent disease and promote good health through different physiological processes, as it is a very effective antioxidant [30]. Furthermore, Stumpf et al. [31] showed that ferulic and *p*-coumaric acids are even able to suppress the growth of fungal pathogens of wheat, such as *Fusarium* species.

#### 2.1.3. Identification, Quantification, and Biological Activity of Alkylresorcinols (ARs, Resorcinolic Lipids)

**Figure 2:**
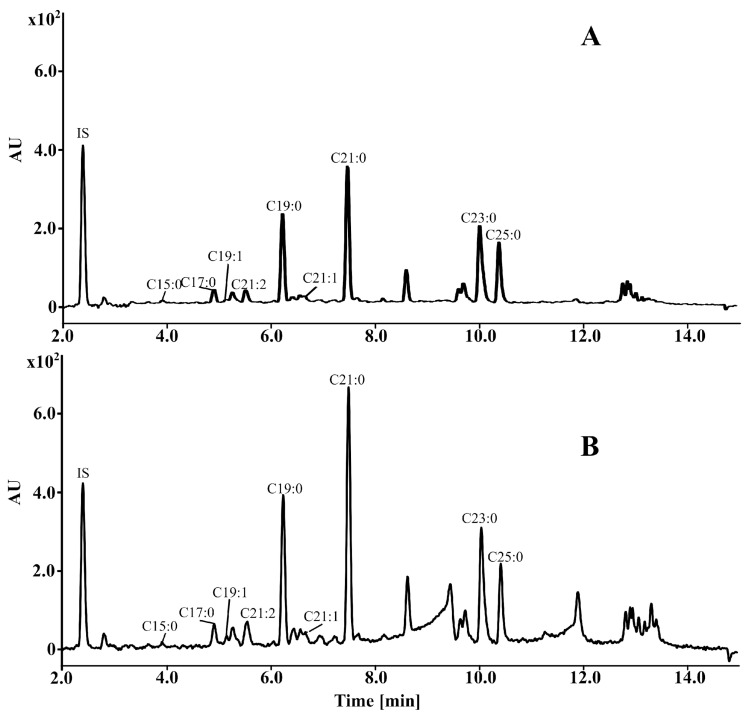
UPLC-PDA chromatograms for all alkylresorcinol derivatives identified in cultivars of winter wheat: 5-*n*-pentadecylresorcinol (C15:0), 5-*n*-heptadecylresorcinol (C17:0), 5-*n*-nonadecenylresorcinol (C19:1), 5-*n*-heneicosadienylresorcinol (C21:2), 5-*n*-nonadecylresorcinol (C19:0), 5-*n*-heneicosenylresorcinol (C21:1), 5-*n*-heneicosylresorcinol (C21:0), 5-*n*-tricosylresorcinol (C23:0), and 5-*n*-pentacosylresorcinol (C25:0); (**A**) cv. Fidelius (*Triticum aestivum* L. subsp. *aestivum*); (**B**) cv. Rokosz (*Triticum aestivum* L. subsp. *spelta*).

Using the UPLC-PDA-MS/MS method, nine AR derivatives were identified in all winter wheat cultivars: 5-*n*-pentadecylresorcinol (C15:0), 5-*n*-heptadecylresorcinol (C17:0), 5-*n*-nonadecenylresorcinol (C19:1), 5-*n*-heneicosadienylresorcinol (C21:2), 5-*n*-nonadecylresorcinol (C19:0), 5-*n*-heneicosenylresorcinol (C21:1), 5-*n*-heneicosylresorcinol (C21:0), 5-*n*-tricosylresorcinol (C23:0), and 5-*n*-pentacosylresorcinol (C25:0) (Figure 2). According to Figure 2 and Table S5, the main homologues in all cultivars were C21:0 and C19:0. The C15:0 and C21:1 derivatives occurred below the limit of quantification (LOQ) in all winter cultivars, while C19:1 occurred below the LOQ in 2017 and in three cultivars in 2018. The total AR content showed significant variability (*p* < 0.05) both between cultivars and across different growth years, ranging from 680.06 μg/g (cv. Pokusa, in 2017) to 1138.12 μg/g (cv. Fidelius, in 2018) (Table S5). The average AR concentration was higher in 2018 than in 2017, at 866.51 and 812.32 µg/g, respectively. In an earlier published study by Kowalska et al. [10], the average AR content in Polish spring wheat grains (cultivated also in 2017–2018) was lower, ranging from 590.96 to 977.43 μg/g in the grain.

The results of the analysis of variance (ANOVA) are summarized in Table S6. The year × cultivar interaction was significant for C19:1, C19:0, C21:0, C25:0 derivatives, total PA, and their activity. A differential effect of year on the content of individual ARs was observed; however, the content of most ARs (except C21:2), the sum of ARs, and their activity were statistically higher in 2018. Cultivars grown in 2018 had significantly higher antioxidant activity (average 0.305 in relation to *α*-tocopherol’s activity) compared to 2017 (mean 0.286). There were no statistically significant differences in the content of C17:0 and C19:0 between the years. The total AR content was highest in cv. Fidelius, both in 2017 and 2018 (mean 1053 µg/g), followed by cv. Belissa (mean 958 µg/g) and cv. KWS Ozon (mean 920 µg/g). These cultivars also showed the highest antioxidant activity (0.371, 0.337, and 0.324, respectively). This may have been due to the high content of C19:0 and C21:0 derivatives, as the phenolic ring length of the AR molecule and the aliphatic side chain strongly influence antioxidant activity. Furthermore, in winter cereals, ARs are indicated as compounds that inhibit the growth and dissemination of fungal infections, and thus their location is compatible with their biological function as antifungal agents. In fact, their amphiphilic nature suggests their role in plant defense [32].

The lowest total content of ARs was shown for cv. Markiza (mean 730 µg/g) and cv. Pokusa (mean 742 µg/g). The highest mean content of C21:0, C19:0, and C23:0 was found in cv. Fidelius; C17:0 was highest in cv. KWS Ozon; C19:1 was highest in cv. Pokusa; C21:2 was highest in cv. Markiza; and C25:0 was highest in cv. Belissa (Table 2). The year effect was significant for C19:1, C21:2, C21:0, C23:0, and C25:0 derivatives, total PA, and their activity. The cultivar was significant for all ARs (with the exception of C15:0 and C21:1 derivatives, which were below the LOQ) and their activity (Table 2). The variability coefficient was low and ranged from 0.00% (for C15:0 and C21:1) to 9.59% (for C25:0).

In previously reported studies by Skrajda-Brdak et al. [33] on winter spelt wheat (*Triticum aestivum* ssp. *spelta*) cultivated in Poland, only five ARs were identified, and their average content was around 723 μg/g. In our research, in cv. Rokosz, nine ARs were identified, and the total AR content was higher (mean 777.64 μg/g). These differences may be due to the use of different analytical methods, cultivars, and environmental conditions.

### 2.2. Comparison of Four Different Farming Systems

#### 2.2.1. Grain Yield, Ear Density, Thousand-Grain Mass, and Colonization of Grain by Fungi

**Figure 3:**
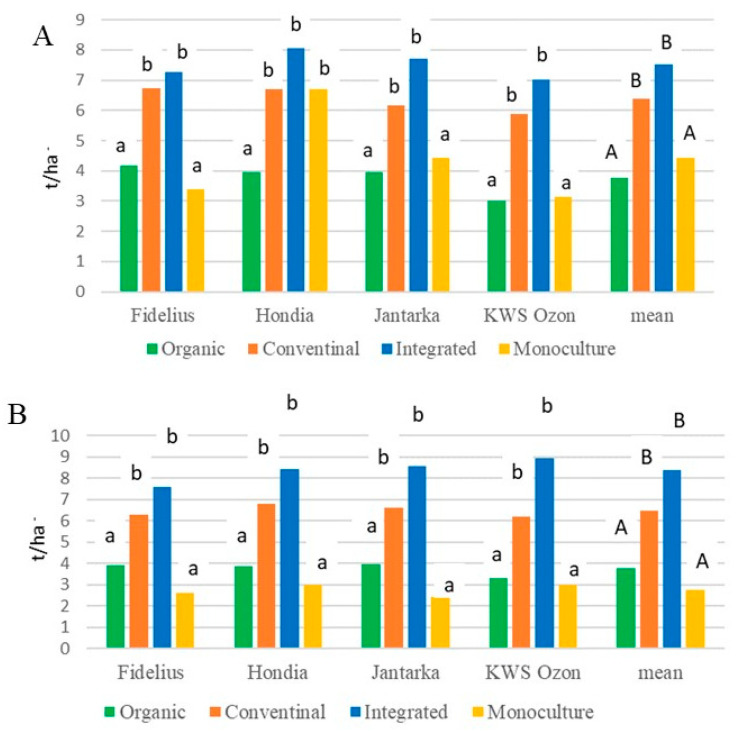
Effect of farming systems on grain yield of 4 winter wheat cultivars in 2-year study (**A**—2017, **B**—2018). Different letters in columns correspond to significant differences between means, according to Tukey’s test at *p* ≤ 0.05.

The highest yield of winter wheat was recorded in the integrated system (on average 7.51 t/ha in 2017 and 8.38 t/ha in 2018), and the lowest in both years of the study occurred in the organic system (on average 3.77 t/ha). The differences in productivity between integrated and conventional systems were statistically insignificant (Figure 3). Wheat yield in the conventional system was lower than in the integrated system by 15–23%, depending on the year. Significant differences in grain yield were also found between organic systems and monoculture. The yield of wheat grain in the organic system was lower than that obtained in the integrated system by 50% in 2017 and 55% in 2018, while in monoculture, the difference was 41% in 2017 and 67% in 2018, respectively.

The wheat crop in the organic farming system was characterized by a significantly lower number of ears and grain maturity compared to the integrated and conventional systems (Figures S1 and S2). The highest number of ears was observed in wheat fields in the conventional system (average 437–475 pcs. m^−2^), and grain maturity was highest in the integrated system (50.1–53.1 g). The reason for the lower grain yield in the organic system was poorer nitrogen nutrition, the occurrence of fungal diseases of leaves in greater intensity, and weed infestation.

No significant interaction between grain yield and its structural elements was found between the production system and cultivars. All evaluated cultivars yielded the highest in the integrated production system. Under organic production conditions, the highest yields were obtained by the following cultivars: Hondia—3.93 t/ha, Jantarka—3.96 t/ha, and Fidelius—4.05 t/ha. The decisive feature for the greater productivity of these cultivars was their ability to produce grain with greater ripeness, averaging 40.1–44.8 g. The results also indicate that out of the four cultivars compared, Hondia exhibits greater tolerance to cultivation in different production systems.

The grains of four winter wheat cultivars cultivated in organic, integrated, conventional, and monoculture systems were infected by *Fusarium* spp. on average in the range of 10.1 to 24.5%, depending on the year. The frequency analysis showed that the largest number of *Fusarium* spp. was isolated from grain from wheat grown in the organic farming system in 2017 (33.1%). In 2017, the percentage of infected grains from organic, conventional, and monoculture production was comparable and amounted to 7.3, 8.6, and 7.6%, respectively. In 2018, statistically insignificant differences in grain colonization were found between the conventional (17.8%) and monoculture (18.4%) systems (Table 3). The cultivar with the lowest percentage of grains infected by *Fusarium* spp. was cv. Fidelius. In both years, significantly more pathogens were isolated from the grain of cv. Jantarka than from other cultivars (in 2017—13.1%, in 2018—34.4%) (Table 3).

#### 2.2.2. Qualitative and Quantitative Analyses and Antioxidant Activity of PA Extracts

A two-year experiment comparing four winter wheat cultivars (Fidelius, Hondia, Jantarka, KWS Ozon) grown under four different crop production systems (ORG, INT, CON, and MONO) was conducted. Significant differences in PA content were found, influenced by cultivar, farming system, and the years of the experiment (Table 4). In our study, we compared as many as four farming systems, whereas in the literature, it is most common to find comparisons of two farming systems, usually ORG and CON [12,19,34,35,36,37,38,39], ORG and INT [40], or three farming systems for wheat, usually ORG, CON, and INT [1,3].

The results of the analysis of variance (ANOVA) are summarized in Table 4. The year × cultivar × system interaction was not significant for total phenolic acid content, nor for the activity and concentration of particular phenolic acids. However, the year × cultivar interaction was significant for caffeic, syringic, sinapic, and salicylic acid (Table S7). The cultivar cv. Jantarka had the highest ARs content in 2017 (cv. Jantarka > cv. Hondia > cv. KWS Ozon > cv. Fidelius), while in 2018, the KWS Ozon cultivar had the highest content (cv. KWS Ozon > cv. Jantarka > cv. Hondia > cv. Fidelius) (Table S7). The year × system interaction was significant for protocatechuic, *p*-OH-benzoic, vanillic, caffeic, syringic, *p*-coumaric, sinapic, and salicylic acid. The highest PA concentrations were seen in the organic farming system in both 2017 and 2018 (Table S8). The cultivar × system interaction was significant for all phenolic acids and their activity. Three cultivars (except for cv. Hondia) showed the highest total PAs content and antioxidant activity in the organic farming system compared to the other systems (ORG > INT > CON > MON) (Table S9). The year effect was significant for protocatechuic, vanillic, syringic, *p*-coumaric, and salicylic acid. Both cultivar and system were significant for all phenolic acids and their activity (Table 4). The variability coefficient was low and ranged from 1.64% (for salicylic acid) to 8.21% (for *p*-OH-benzoic acid).

Considering the effect of the cultivation system on the content of phenolic acids over different years, it was shown that the organic system had statistically higher contents of the tested compounds, regardless of the year of the study (Table S8). Taking into account the effect of the farming system on the four wheat cultivars, all cultivars grown in the organic system exhibited significantly higher levels of protocatechuic, *p*-OH-benzoic, caffeic, and ferulic acids, as well as total phenolic acid content and antiradical activity (Table S9).

The highest total phenolic acid content was found in cv. KWS Ozon (906 µg/g) (Table 5). Regarding the effect of farming systems, the concentrations of most phenolic acids (except for syringic and salicylic acid), as well as the total phenolic acid content (956 µg/g) and their activity (0.204), were higher in organic wheat grains than in other farming systems (ORG > INT > CON > MONO). The differences observed were statistically significant (*p* < 0.001). The reason for this may be that increased PA content is an important defense factor for wheat growing under stress conditions, exposed to attacks by insects, pathogens, and herbivores, as well as subjected to the less selective herbicide treatments used in organic farming systems [41]. The results of Buczek et al. [1] also showed that the ORG farming system, compared to the INT and CON systems, resulted in a higher increase in total phenolic acids, especially ferulic, vanillic, and syringic acid, in winter wheat grains cultivated from 2015 to 2018 in southeastern Poland. A study by Żuchowski et al. [19] showed that grains of winter wheat cultivars from the ORG system had significantly more total phenolic acids than wheat cultivated in the CON system, by 5.0 µg/g. Similar observations were made by Zrcková et al. [38], who found statistically significant differences between the ORG and CON systems in the content of antioxidant compounds, such as phenolic acid content (797.0 and 744.7 µg/g, respectively). The higher phenolic acid content of the grain in the ORG system may be due to variations in plant metabolism owing to differences in soil nitrogen availability in the ORG and CON systems (carbon/nutrient balance hypothesis) [19,42].

Czaban et al. [3] determined the PA concentration in the grain of four winter wheat cultivars grown in Poland in 2010 under three cropping systems—CON, INT, and ORG. They showed that both winter wheat cultivars and crop production methods influenced phenolic acid content. Wheat grain cultivated with conventional farming technology contained the most phenolic acids. In our research, the highest content of total PAs was found for cultivation in ORG (955.6 µg/g), and the lowest content was found for cultivation in monoculture (828.4 µg/g) (Table 5). FER was also the main phenolic acid in all samples. Average FER levels ranged from 78.3% (cv. KWS Ozon, 2017) to 85.8% (cv. Hondia, 2018) of total phenolic acid content. Lower contents of this compound were detected by Żuchowski et al. [35] in Polish winter wheat cultivars grown under two farming systems (ORG and CON). There was a significant positive correlation between total phenolic acids and total antiradical activity (Table S2). Differences were observed in the antioxidant activity of winter wheat grain between the farming systems. Comparing the four farming systems, grains in the organic system showed the highest antioxidant activity. For the other three cropping systems, the antioxidant activity was not statistically significant (Table S2). Zrcková et al. [38] showed that in organic cultivation, wheat grain had higher antioxidant levels, whereas the variability of antioxidant content in winter wheat grain depended on the cultivar, weather conditions, and the cultivation system.

#### 2.2.3. Characterization, Quantification, and Antiradical Activity of AR Extracts

Significant differences in ARs content were influenced by the years of the experiment, cultivar, and farming system (with the exception of C15:0 and C21:1 derivatives, which were below the LOQ) (Table 6). The results of the analysis of variance (ANOVA) are summarized in Table 7. The year × cultivar × system interaction was not significant for C25:0. The year × cultivar interaction was not significant for C23:0 and C25:0. cv. Fidelius had the highest total ARs content (mean 875.3 µg/g) compared to the other cultivars in both cultivation years (cv. Fidelius > cv. KWS Ozon > cv. Hondia > cv. Jantarka) (Table S10).

The year × system interaction was significant for C19:1, C21:2, C21:0, and C25:0, total ARs, and their activity. The highest total AR concentrations were seen for the organic farming system in both 2017 and 2018, at 885.91 and 910.03 µg/g, respectively (Table S11). The limitation in the use of synthetic pesticides favors the production of plant-specific substances, which are a natural response to pathogen attacks due to the inducing effects of environmental pressure. The cultivar × system interaction was significant for C19:1, C21:2, C19:0, C21:0, and C23:0, total ARs, and their activity. All cultivars showed the highest total AR content and antioxidant activity in the organic farming system (mean 898.0 µg/g and 0.314, respectively), compared to the other systems (ORG > MON > CON > INT) (Table S12). Among the tested cultivars, the grain of cv. Fidelius showed the highest activity (0.368) in the ORG system. The results of this research indicated that the antiradical activity of wheat extracts had a positive correlation with the total amount of ARs. The variability coefficient was low and ranged from 0.00% (for C15:0 and C21:1) to 10.3% (for C23:0) (Table 7).

In an earlier study, Kowalska et al. [43] determined the profile of ARs in *T. aestivum* winter wheat cultivars grown in Poland under two different production systems: CON and ORG. The profile of ARs included five 5-*n*-alkylresorcinol derivatives. The total content of resorcinolic lipids ranged from 203 to 1272 μg/g (mean 796 μg/g). Organically grown cultivars had a total AR content significantly higher than that of conventionally grown cultivars. Organic farming is especially attractive for eco-friendly, healthy food, as the use of conventional synthetic chemicals is reduced. Ecological grains tend to have a lower content of macronutrients but a higher level of plant metabolites.

## 3. Materials and Methods

### 3.1. Plant Material

This study used data from an experiment in which the grain quality of 12 winter wheat cultivars was evaluated from 2 winter wheat species: common wheat, *Triticum aestivum* L. subsp. *aestivum* (cv. Arktis, Belissa, Estivus, Fidelius, Hondia, Jantarka, KWS Ozon, Linus, Markiza, Ostka Strzelecka, Pokusa), and spelt wheat, *Triticum aestivum* L. subsp. *spelta* (L.) Thell. (cv. Rokosz). All cultivars are listed in the Common Catalog of Varieties of Agricultural Plant Species. Cultivars were selected for this study using selection criteria that included: quality criterion (the selected varieties are among the wheat with the best quality parameters), morphological differentiation, above-average resistance to fungal pathogens, frost resistance, and a large share in the seed market. The experiment was established using the randomized sub-block method in four replicates. The factors of the experiment were cultivars (12 cultivars) and the year of grain harvest (2017, 2018) in the organic production system. The second experiment involved comparing the productivity of four winter wheat cultivars (Fidelius, Hondia, Jantarka, KWS Ozon) under four different farming systems, ORG, INT, CON, and MONO, over two years.

### 3.2. Characteristics of the Experimental Plots Under Different Farming Systems

**Figure 4:**
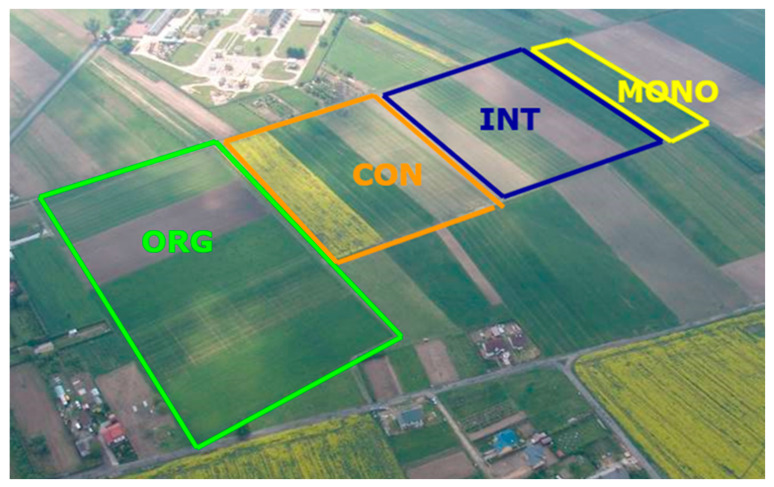
Scheme of experimental fields with different farming systems (fot. K. Jończyk). ORG = organic, INT = integrated, CON = conventional, and MONO = monoculture farming system.

This study was conducted from 2017 to 2018 at the Agricultural Research Station of the Institute of Soil Science and Plant Cultivation-State Research Institute (IUNG-PIB) in Osiny, Poland (N: 51°28′, E: 22°30′), as part of a long-term experiment (initiated in 1994) comparing four different farming systems: ORG, CON, INT, and MONO (Figure 4).

In the organic system (potato, spring wheat, red clover with grass grown, winter wheat + catch crop, and mixed oats with vetch) neither mineral fertilization nor pesticides were applied. Organic fertilization included only manure application (30 t/ha) before the potato crop. The conventional system (winter rape, winter wheat, and spring wheat) was conducted as an intensive crop production technology, whereas the integrated system (potato, spring wheat, faba bean, and winter wheat + catch crop) represented a more extensive conventional technology. Winter wheat monoculture (MONO) was an extreme example of simplification in the management method. Intensive production technology aimed at reducing the adverse effects of continuous wheat cultivation was used. Organic fertilization, in the form of straw plowed, was performed every other year (Table S13). The area of each field in all systems was about 1 ha. The soil was Haplic Luvisol with a loamy sand texture. The annual mean temperature and precipitation at the site are 8.3 °C and 518 mm, respectively. The soil pH (in 1 M KCl) ranged from 5.6 to 5.9 in each system. The content of basic nutrients in the soil corresponded in each system, ORG, CON, INT, and MONO, to the following values: total carbon (8.6; 8.1; 8.4; 8.0 g/kg); phosphorus (45.5; 68.2; 72.1; 65.8 mg/kg); potassium (124.5; 205.5; 220.4; 198.7 mg/kg); and magnesium (54.5; 51.2; 58.7; 52.7 mg/kg). These data were taken from the topsoil (0–30 cm depth). Soil tillage was similar in all crop production systems and used a traditional moldboard plowing system.

### 3.3. Meteorological Conditions

The course of weather conditions was described on the basis of average monthly values of air temperature and precipitation totals, in comparison with averages from the multi-year period of 1950–2010. Meteorological data were obtained from agrometeorological stations located near the experimental fields in Osiny. In the autumn of 2016, rainfall deficiencies were recorded, which made it difficult to carry out sowing at the optimal time and perform tillage treatments. In addition, periodic temperature drops to −18 to −22 °C were recorded in January, causing plant damage and thinning of the canes. The summer months saw a deficit in precipitation, particularly severe in June. Moisture deficits occurring during critical periods for wheat development resulted in a reduction in production shoots and insufficient grain formation. In the 2017/2018 season, the period immediately preceding sowing was characterized by increased rainfall, which contributed to delays in sowing and difficulties in pre-sowing tillage. During the winter period, air temperatures were generally higher than the multi-year average (Figure S3). Plants survived the winter period in good condition, and no major damage was observed. Since the beginning of vegetation in 2018, precipitation levels were close to the multi-year average. During the months of May–June, there were rainfall deficiencies that slowed down the development of wheat, affecting shoot reduction and grain filling.

### 3.4. Biometric Analyses, Grain Yield, and Assessment of Plant Infestation by Pathogens

The number of ears, thousand-kernel weight, grain yield, and plant infestation by pathogens were determined using the method previously published by Kowalska et al. [10].

### 3.5. Assessment of Fusarium spp. Occurrence

In order to determine the fungi inhabiting wheat kernels, a mycological analysis was performed. After the harvest, the grains were subjected to the mycological analysis. From each combination, 4 × 100 grains were randomly selected. After rinsing with running water and decontaminating for 2.5 min in 1% NaOCl, followed by rinsing three times with sterile water, the grains were placed into BD DIFCO™ Potato Dextrose Agar (PDA; Becton, Dickinson and Company, Franklin Lakes, NJ, USA) (pH = 5.5), where they were incubated for 6 days at a temperature of 20 °C. The fungus-growing colonies were subsequently grafted onto PDA slants, where they were identified using mycological keys [44].

### 3.6. Reagents

LC-MS-grade methanol and acetone were purchased from Merck (Darmstadt, Germany). Formic acid of LC-MS grade was also supplied by Merck (Darmstadt, Germany). The following reference standards were purchased: 4-dodecylresorcinol and 5-heneicosylresorcinol from Sigma-Aldrich (St. Louis, MO, USA, Milwaukee, WI, USA) and m-hydroxybenzoic acid from POCH S.A. (Gliwice, Poland). Acetone, n-hexane, ethyl acetate, and 2-propanol were imported from Fisher Chemical (Bremen, Germany). Solid reagents, sodium hydroxide and ascorbic acid, were purchased from Sigma-Aldrich (St. Louis, MO, USA, Milwaukee, WI, USA). Hydrochloric acid solution (35–38%) was imported from Chempur (Pieczary Śląskie, Poland). Ultrapure water was obtained in-house using a purification system (Milli-Q-Simplicity-185, Millipore Corp., Molsheim, France).

### 3.7. Phenolic Acid Analysis

#### 3.7.1. Extraction of PAs from Winter Wheat Cultivars

A Soxhlet apparatus with n-hexane was used to degrease the ground material. Each sample was prepared following the modified method of Żuchowski et al. [35]. All samples were subjected to hydrolysis with 3 mL of 4 M NaOH and 3 mL of a 2% ascorbic acid solution for 4 h at room temperature. An internal standard of 20 μg m-hydroxybenzoic acid was added to the process. After hydrolysis, samples were cooled on ice and acidified using ice-cold 6M HCl to achieve a pH of about 2. The obtained preparations were centrifuged at 8000 rpm (Sigma 2–16) for 20 min. The supernatants were extracted thrice with ethyl acetate. The organic phase was collected, filtered, and evaporated to dryness on a rotary evaporator at 35 °C. The extracts were dissolved in 30% methanol, transferred to 2 mL Eppendorf, and stored in a freezer at approximately −20 °C.

#### 3.7.2. UPLC Conditions for Quantitative Analysis of PAs

An ACQUITY UPLC system equipped with a PDA and a triple quadrupole mass detector (TQD, Waters, Milford, MA, USA) was used to determine phenolic acids from the obtained hydrolysates. The separation of each sample was performed on a Waters Acquity UPLC HSS C18 column (100 × 2.1 mm, 1.8 µm) at 30 °C. The mobile phase consisted of solvent A (acidified water, 0.1% formic acid) and solvent B (acidified acetonitrile, 0.1% formic acid). The gradient of the solvents was programmed as follows: 8% to 20% B in 10.6 min; 20% to 95% B in 2.9 min; and 95% to 8% B in 2 min. The sample injection volume was 2.5 μL, and the flow rate was 0.45 mL/min. All compound interpretation was based on data from UV and MS spectra. Data processing was carried out using the MassLynx V4.2 software package (Waters Corp., Milford, MA, USA).

#### 3.7.3. Quantification of Individual PAs

Phenolic acids were detected using a multiple reaction monitoring (MRM) scan in negative ionization mode. The concentrations of the identified compounds (μg/mg of the grain) were calculated based on calibration curves (Table S14).

#### 3.7.4. Antiradical Activity of PA Extracts

The antioxidant activity of PA extracts in winter wheat cultivars was measured using a benchtop thin-layer chromatography−2,2-diphenyl-1-picrylhydrazyl radical (TLC-DPPH^•^) bioassay. The results of the TLC–DPPH^•^ test were documented by flat-bed scanning, saved in the form of jpg documents, and further processed by means of an open-source program ImageJ, developed at the National Institute of Health in the USA (ImageJ 1.48v; Java 1.6.0_20). This method was previously described by Kowalska et al. [29].

### 3.8. Alkylresorcinol Analysis

#### 3.8.1. Extraction of ARs from Winter Wheat

The extraction of alkyloresorcinols from winter wheat was carried out according to Kowalska et al. [29] with minor modifications. Two grams of milled, non-defatted material were extracted with 40 mL of acetone. Before extraction, 20 µL of a 4 mg/mL 4-dodecylresorcinol internal standard solution was added to each sample. After a 24 h extraction process with multiple sonications (four times), the extracts were centrifuged at 11,000 rpm^−1^ for 5 min. The supernatant was collected and evaporated to dryness under reduced pressure. The samples in the flasks were dissolved in 2 mL of 2-propanol and quantitatively transferred to Eppendorf. The prepared samples were stored in a freezer at approximately −20 °C.

#### 3.8.2. UPLC Conditions for Quantitative Analysis

The samples of winter wheat were analyzed using the ACQUITY UPLC system, equipped with a PDA and a triple quadrupole mass detector (TQD, Waters, Milford, MA, USA). The separation of compounds was achieved using an ACQUITY BEH C8 (100 × 2.1 mm, 1.8 μm; Waters) column, which was maintained at 50 °C. The gradient mobile phase consisted of solvent B (methanol with 0.1% formic acid) and solvent A (water Milli-Q with 0.1% formic acid): 0.00–1.00 min, 75% B; 1.00–12.00 min, 75–96% B; 12.00–12.10 min, 96–100% B; 12.10–14.00 min, 100%; 14.00–14.05 min, 100–75% B; and 14.05–16.00 min, 75% B, at a flow rate of 0.50 mL/min. The injection volume was 2.5 μL. Analyte peaks were interpreted based on the UV spectra. The PDA operated in the range of 210–450 nm, and the resolution was set at 3.6 nm. Data were processed using Waters MassLynx 4.2 software (Waters Corp., Milford, MA, USA).

#### 3.8.3. Quantification of Individual ARs

A quantitative analysis of the ARs was performed based on data from UV spectra at a wavelength of 275 nm. The quantification of individual analytes was based on the method of internal standard (4-dodecylresorcinol) and peak area calculated from UV chromatograms. A 5-heneicosylresorcinol was used as a group standard. The calibration curve (y = 21.774x − 1.5669) was prepared with 8 concentrations and showed a linear response (R^2^ = 0.9998) within the tested range (2.5–250.0 μg/mL).

#### 3.8.4. Antioxidant Activity of ARs Extracts

The antiradical activity of AR extracts in winter wheat cultivars was established using the TLC-DPPH^•^ method with the ImageJ program. This method was first reported in 2020 by Kowalska and Jędrejek [43].

### 3.9. Statistical Analysis

The obtained results were subjected to statistical evaluation using Statistical versions 10 and 12 (Stat. Soft. Inc., Tulsa, OK, USA) software based on the strength of multivariate analysis of variance and post hoc analysis. Analysis of variance (ANOVA) followed by Tukey’s test, with a significance level set at *p* < 0.05, were used to determine significant differences [45]. The experiments were conducted in triplicate (n ≥ 3), and the results were presented as mean values ± standard deviation (SD). Statistical calculations for the comparison of the abundance of grains colonized by *Fusarium* spp. were made by means of a frequency analysis—chi-squared (χ^2^) test of concordance.

## 4. Conclusions

To the best of our knowledge, this is the first study to describe the effect of four different framing systems, among other factors, on the content of bioactive compounds in winter wheat cultivars. Nine phenolic acids (mainly ferulic, *p*-coumaric, and sinapic acids), nine alkylresorcinols (main homologues—C21:0 and C19:0), and their antiradical activity were identified and determined. Despite the low yield (1.89 t/ha in 2017), the *T. aestivum* L. subsp. *aestivum*—cv. Rokosz had the highest total phenolic acid content (mean 1302.3 µg/g). Ferulic acid was the main phenolic acid in all samples. The other phenolic acids were present in lower concentrations and can be divided as follows: FER > PCO > SIN > CAF > VAN > SYR > PRO > POH > SAL. In contrast, *T. aestivum* L. subsp. *aestivum*—cv. Fidelius had the highest yield (4.17 t/ha in 2017), resistance to *Fusarium* fungi, alkylresorcinol content (mean 1053 µg/g), and antioxidant capacity (0.371). In addition, the influence of the farming system (which also included monoculture) and environmental conditions in the year were evaluated. The concentrations of most phenolic acids (except for syringic and salicylic acid), as well as the total phenolic acid content (956 µg/g) and their activity (0.204), were higher in organic wheat grains than in other framing systems (ORG > INT > CON > MONO). All cultivars showed the highest total AR content and antioxidant activity in the organic farming system (mean 898.0 µg/g and 0.314, respectively), compared to the other systems (ORG > MON > CON > INT). These results are favorable for future wheat production, which aims to produce wheat grains enriched with natural antioxidants.

## Figures and Tables

**Table 1:** Influence of the year and cultivar on the content and antioxidant activity of phenolic acids. Mean phenolic acids (µg/g of the grain), total phenolic acid concentration (µg/g of the grain), and antiradical activity (in relation to caffeic acid’s activity = 1.00) of twelve winter wheat cultivars, cultivated in an organic farming system, over two years.

**Phenolic Acid**	**Year**		**Cultivar**				
	**2017**	**2018**	**Fidelius**	**Hondia**	**Jantarka**	**KWS Ozon**	**Arktis**
Protocatechuic acid	5.89 ±0.14 a	5.19 ±0.11 b	5.90 ±0.25 b	4.71 ±0.13 de	4.92 ±0.22 de	5.32 ±0.20 b–d	5.57 ±0.16 bc
*p*-OH-Benzoic acid	3.17 ±0.30 b	3.35 ±0.24 a	2.03 ±0.11 g	2.15 ±0.09 fg	2.11 ±0.10 fg	2.73 ±0.09 ef	2.20 ±0.12 e–g
Vanillic acid	26.02 ±1.18 a	24.37 ± 0.78 b	24.13 ±0.68 c–e	21.74 ±0.50 ef	20.44 ±0.52 f	24.73 ±0.71 cd	23.09 ±0.65 c–e
Caffeic acid	38.88 ±1.80 a	33.98 ±0.89 b	31.16 ±0.80 de	26.83 ±0.6 e	37.72 ±0.58 a–d	44.97 ±8.46 a	40.70 ±1.43 a–c
Syringic acid	14.54 ±0.74 b	14.98 ±0.67 a	16.02 ±0.32 bc	15.91 ±0.35 bc	11.40 ±0.05 hi	13.99 ±0.27 de	11.13 ±0.22 i
*p*-Coumaric acid	65.87a ±13.05 a	57.04b ±7.89 b	47.56 ±4.43 b	34.28 a ±2.22 b	37.51 ±2.38 b	50.33 ±4.39 b	36.07 ±1.24 b
Ferulic acid	829.01 ±13.19 a	826.28 ±14.78 a	712.77 ±23.40 e	757.30 ±17.41 de	824.73 ±16.69 b–d	851.01 ±23.60 a–c	795.34 ±9.46 c–e
Sinapic acid	44.61 ±1.32 b	45.90 ±1.68 a	38.08 ±0.84 fg	39.50 ±0.80 ef	57.40 ±1.14 b	46.79 ±2.00 cd	41.89 ±0.66 d–f
Salicylic acid	1.63 ±0.02 b	1.68 ±0.02 a	1.54 ±0.01 e	1.53 ±0.02 e	1.55 ±0.02 e	1.66 ±0.04 cd	1.62 ±0.05 d
Total	1029.63 ±24.49 a	1012.78 ±19.40 a	879.19 ±24.31 f	903.96 ±20.54 ef	997.78 ±20.34 c–e	1041.53 ±25.13 b–d	957.62 ±11.03 d–f
Antiradical acitvity	0.220 ±0.005 a	0.217 ±0.004 a	0.188 ±0.005 f	0.193 ±0.004 e	0.213 ±0.004 c–e	0.223 ±0.005 bc	0.205 ±0.002 de
**Phenolic Acid**	**Cultivar**						
	**Belissa**	**Estivus**	**Linus**	**Markiza**	**Ostka Strzelecka**	**Pokusa**	**Rokosz**
Protocatechuic acid	6.77 ±0.13 a	5.53 ±0.28 bc	5.15 ±0.03 c–e	5.53 ±0.14 bc	5.68 ±0.28 bc	4.64 ±0.11 e	6.74 ±0.45 a
*p*-OH-Benzoic acid	5.36 ±0.18 b	2.78 ±0.23 e	3.74 ±0.28 d	4.56 ±0.19 c	2.39 ±0.14 e–g	2.08 ±0.15 g	7.02 ±0.33 a
Vanillic acid	23.91 ±0.36 c–e	22.75 ±0.39 c–e	25.00 ±1.31 c	27.84 ±0.46 b	24.01 ±0.63 c–e	22.05 ±0.50 d–f	42.68 ±2.58 a
Caffeic acid	34.91 ±1.15 b–e	36.23 ±1.71 a–e	40.87 ±2.16 a–c	33.10 ±0.97 c–e	37.56 ±2.43 a–d	29.27 ±0.74 de	43.85 ±4.67 ab
Syringic acid	12.74 ±0.87 f–g	13.26 ±0.35 ef	14.93 ±0.42 cd	26.73 ±0.59 a	12.12 ±0.22 g–i	11.76 ±0.31 g–i	17.15 ±0.87 b
*p*-Coumaric acid	41.18 ±3.97 b	37.56 ±2.90 b	48.51 ±0.67 b	41.63 ±3.04 b	49.43 ±3.46 b	48.48 ±1.76 b	264.94 ±25.88 a
Ferulic acid	830.61 ±14.81 b–d	858.14 ±16.75 a–c	929.64 ±26.73 a	905.40 ±26.51 ab	872.42 ±23.89 a–c	710.39 ±20.68 e	883.97 ±24.24 a–c
Sinapic acid	44.68 ±1.63 c–e	48.59 ±0.77 c	65.47 ±3.07 a	43.14 ±1.96 d–f	39.50 ±1.20 ef	43.90 ±1.01 c–e	34.09 ±0.70 g
Salicylic acid	1.64 ±0.04 cd	1.63 ±0.03 d	1.75 ±0.01 b	1.68 ±0.03 cd	1.70 ±0.03 bc	1.67 ±0.04 cd	1.92 ±0.03 a
Total	1001.91 ±19.14 c–e	1026.50 ±19.14 b–d	1135.07 ±31.96 b	1089.60 ±29.92 bc	1044.82 ±30.03 b–d	874.24 ±23.25 f	1302.35 ±57.02 a
Antiradical acitvity	0.214 ±0.004 cd	0.220 ±0.004 bc	0.243 ±0.007 b	0.233 ±0.006 b	0.223 ±0.006 b–d	0.187 ±0.005 f	0.279 ±0.012 a

Different letters indicate significant differences (*p* < 0.05) according to the Tukey test. Values within each acid were compared separately.

**Table 2:** Influence of the year and cultivar on the content and antioxidant activity of ARs. Mean ARs (µg/g of the grain), total phenolic acid concentration (µg/g of the grain), and antiradical activity (in relation to caffeic acid’s activity = 1.00) of twelve winter wheat cultivars, cultivated in organic farming system, over two years.

**Alkyl-** **Resorcinol**	**Year**		**Cultivar**				
	**2017**	**2018**	**Fidelius**	**Hondia**	**Jantarka**	**KWS Ozon**	**Arktis**
C15:0	LOQ	LOQ	LOQ	LOQ	LOQ	LOQ	LOQ
C17:0	28.31 ± 1.14 a	28.12 ±1.02 a	36.32 ±3.45 ab	28.51 ±1.21 a–d	19.35 ±0.62 e	37.20 ±1.61 a	22.38 ±1.65 de
C19:1	LOQ	22.56 ±2.41 a	11.11 ±5.04 ef	LOQ	12.93 ±5.84 d–f	LOQ	10.85 ±5.04 f
C21:2	42.57 ±1.63 a	37.54 ±1.26 b	36.12 ±4.03 b–d	41.16 ±3.60 a–d	46.00 ±1.51 ab	46.25 ±2.47 ab	22.50 ±1.59 e
C19:0	221.48 ±7.33 a	224.60 ±6.54 a	289.68 ±11.94 a	228.83 ±6.80 cd	161.26 ±3.16 g	259.34 ±7.77 b	201.43 ±2.40 ef
C21:1	LOQ	LOQ	LOQ	LOQ	LOQ	LOQ	LOQ
C21:0	405.83 ±9.20 b	421.29 ±8.75 a	507.50 ±21.51 a	452.97 ±12.47 b	385.18 ±8.10 d–f	448.84 ±14.38 b	421.19 ±1.79 b–d
C23:0	87.13 ±2.37 b	96.96 ±2.46 a	122.73 ±8.04 a	89.82 ±7.75 b	88.86 ±2.52 b	95.79 ±2.79 b	95.02 ±0.92 b
C25:0	27.01 ±2.52 b	35.45 ±2.15 a	40.66 ±4.54 ab	32.97 ±3.28 ab	30.64 ±2.63 bc	32.88 ±1.14 b	37.07 ±6.07 ab
Total	812.32 ±18.43 b	866.51 ±17.90 a	1053.11 ±46.89 a	874.24 ±24.09 b–d	744.22 ±15.29 fg	920.31 ±29.12 bc	810.45 ±6.61 d–g
Antiradical acitvity	0.286 ±0.006 b	0.305 ±0.006 a	0.371 ±0.017 a	0.308 ±0.008 bc	0.262 ±0.005 f	0.324 ±0.010 bc	0.286 ±0.002 fg
**Alkyl-** **Resorcinol**	**Cultivar**						
	**Belissa**	**Estivus**	**Linus**	**Markiza**	**Ostka Strzelecka**	**Pokusa**	**Rokosz**
C15:0	LOQ	LOQ	LOQ	LOQ	LOQ	LOQ	LOQ
C17:0	32.92 ± 2.01 a–c	25.85 ±0.72 c–e	30.41 ±3.05 a–d	24.89 ±0.55 c–e	25.98 ±0.98 c–e	28.28 ±0.47 b–d	26.48 ±1.21 c–e
C19:1	18.76 ± 8.39 ab	13.90 ± 6.24 c–f	17.33 ±7.79 a–c	LOQ	14.98 ±6.80 b–e	20.31 ±9.10 a	15.22 ±6.86 b–d
C21:2	35.03 ±1.72 cd	46.72 ±0.98 a	33.13 ±3.52 d	48.94 ±2.16 a	41.64 ±1.60 a–d	44.62 ±2.11 a–c	38.52 ±1.48 a–d
C19:0	269.11 ±8.05 b	196.93 ±5.66 ef	249.02 ±13.33 bc	185.79 ±2.48 f	212.16 ±7.02 de	201.80 ±3.86 ef	212.08 ±8.26 de
C21:1	LOQ	LOQ	LOQ	LOQ	LOQ	LOQ	LOQ
C21:0	459.76 ±8.92 b	434.72 ±14.72 bc	378.23 ±8.18 ef	349.76 ±5.57 f	372.68 ±10.42 ef	352.91 ±8.43 f	398.99 ±16.04 c–e
C23:0	95.03 ±5.75 b	94.55 ±3.78 b	88.31 ±7.72 b	83.60 ±2.30 b	87.18 ±3.07 b	77.26 ±2.54 b	86.36 ±2.87 b
C25:0	46.91 ±3.36 a	29.00 ±2.64 bc	31.32 ±3.11 b	37.34 ±2.22 ab	39.17 ±2.21 ab	16.77 ±7.50 c	LOQ
Total	957.51 ±16.46 b	841.66 ±32.84 c–e	827.75 ±17.92 c–f	730.32 ±11.03 f	793.81 ±29.05 d–g	741.96 ±28.99 fg	777.64 ±34.01 e–g
Antiradical acitvity	0.337 ±0.006 b	0.297 ±0.012 cd	0.292 ±0.006 ef	0.257 ±0.004 f	0.280 ±0.010 ef	0.261 ±0.010 g	0.274 ±0.012 e–g

LOQ—below the limit of quantification. Different letters indicate significant differences (*p* < 0.05) according to the Tukey test. Values within each alkylresorcinol were compared separately.

**Table 3:** *Fusarium* fungi (% of colonized grains) isolated from winter wheat grains, grown in different farming systems, from 2017 to 2018.

Cultivar	Farming System				Average for a Cultivar
	ORG	INT	CON	MONO	
2017					
Fidelius	5.0 b ^1^ B	17.0 b A	5.7 b B	5.0 a B	8.2 c
Hondia	8.3 ab B	23.1 ab A	3.5 b C	8.5 a B	10.9 ab
Jantarka	6.2 ab C	24.0 a A	13.6 a B	8.5 a C	13.1 a
KWS Ozon	9.8 a A	10.7 c A	11.5 a A	8.2 a A	10.1 bc
Mean	7.3 B ^2^	18.7 A	8.6 B	7.6 B	10.5
2018					
Fidelius	18.5 b B	36.0 a A	9.5 b C	14.0 b BC	19.5 c
Hondia	24.0 b A	18.5 c A	5.0 b C	12.5 b B	15.0 d
Jantarka	46.5 a A	28.0 b C	27.0 a C	36.0 a B	34.4 a
KWS Ozon	43.5 a A	33.0 ab B	29.5 a B	11.0 b C	29.3 b
Mean	33.1 A	28.9 B	17.8 C	18.4 C	24.5

^1^ Values marked with different lowercase letters indicate a significant difference between cultivars. ^2^ Values marked with different capital letters indicate a significant difference between farming systems.

**Table 4:** Summary of analysis of variance results. Significance of effect (years, cultivars, and systems) in the analysis of variance and variability coefficient of phenolic acids.

Phenolic Acid	Source of Variability							V (%) ^#^
	Year (Y)	Cultivar (C)	System (S)	Y × C	Y × S	C × S	Y × C × S	
Protocatechuic acid	***	***	***	ns	***	***	ns	5.95
*p*-OH-Benzoic acid	ns	***	***	ns	***	***	ns	8.21
Vanillic acid	***	***	***	ns	**	***	ns	4.29
Caffeic acid	ns	***	***	***	***	***	ns	7.74
Syringic acid	***	***	***	**	***	***	ns	5.05
*p*-Coumaric acid	***	***	***	***	***	***	ns	7.44
Ferulic acid	ns	***	***	ns	ns	***	ns	5.85
Sinapic acid	ns	***	***	**	**	***	ns	5.67
Salicylic acid	***	***	**	**	***	***	ns	1.64
Total	ns	***	***	ns	ns	***	ns	5.78
Antiradical acitvity	ns	***	***	ns	ns	***	ns	5.93

Significance levels are *** *p* < 0.001, ** *p* < 0.01, ns—not significant; ^#^ Variability coefficient V (%) = √S^2^/x × 100%.

**Table 5:** Influence of the year, cultivar, and farming system on the content and antioxidant activity of phenolic acids. Mean phenolic acids (µg/g of the grain), total phenolic acid concentration (µg/g of the grain), and antiradical activity (in relation to caffeic acid’s activity = 1.00) of four winter wheat cultivars, cultivated in four farming system, over two years.

Phenolic Acid	Year		Cultivar				System			
	2017	2018	Fidelius	Hondia	Jantarka	KWS Ozon	ORG	CON	INT	MONO
Protocatechuic acid	4.49 ±0.11 a	3.91 ±0.10 b	4.64 ±0.17 a	4.13 ±0.16 b	3.99 ±0.14 b	4.03 ±0.18 b	5.21 ±0.13 a	3.75 ±0.09 c	3.98 ±0.11 b	3.87 ±0.08 b
*p*-OH-Benzoic acid	1.88 ±0.06 a	1.92 ±0.08 a	1.58 ±0.11 c	1.99 ±0.06 b	1.71 ±0.09 c	2.33 ±0.07 a	2.25 ±0.07 a	1.59 ±0.10 c	1.73 ±0.11 c	2.05 ±0.07 b
Vanillic acid	22.16 ±0.34 a	21.17 ±0.31 b	23.82 ±0.33 a	22.05 ±0.26 c	19.19 ±0.26 b	21.60 ±0.45 c	22.76 ±0.46 a	21.32 ±0.39 bc	20.85 ±0.40 c	21.73 ±0.55 b
Caffeic acid	24.25 ±1.70 a	22.76 ±0.85 a	21.71 ±1.23 b	20.92 ±0.83 b	26.36 ±1.43 a	25.03 ±3.11 a	35.17 ±2.45 a	19.66 ±0.40 b	19.73 ±0.59 b	19.46 ±0.62 b
Syringic acid	15.34 ±0.34 a	14.77 ±0.39 b	16.74 ±0.22 b	17.55 ±0.29 a	11.71 ±0.21 d	14.22 ±0.17 c	14.33 ±0.41 b	15.61 ±0.56 a	15.03 ±0.55 a	15.26 ±0.55 a
*p*-Coumaric acid	35.83 ±1.48 a	26.22 ±1.75 b	27.26 ±2.76 b	34.28 ±2.41 a	30.86 ±1.98 ab	31.69 ±2.65 a	42.42 ±2.15 a	26.28 ±1.94 b	26.12 ±1.73 b	29.27 ±2.51 b
Ferulic acid	726.23 ±10.14 a	722.98 ±10.43 a	671.22 ±11.46 b	739.00 ±12.30 a	728.97 ±13.70 a	759.23 ±14.46 a	786.45 ±14.87 a	704.61 ±13.44 b	713.30 ±14.33 b	694.06 ±5.36 b
Sinapic acid	43.61 ±1.07 a	44.26 ±1.13 a	34.95 ±0.56 d	42.00 ±0.78 c	52.76 ±1.01 a	46.02 ±0.89 b	45.44 ±1.70 a	42.90 ±1.52 b	46.22 ±1.74 a	41.17 ±1.01 b
Salicylic acid	1.53 ±0.01 b	1.64 ±0.01 a	1.56 ±0.02 c	1.60 ±0.02 b	1.57 ±0.02 c	1.62 ±0.02 a	1.57 ±0.02 b	1.60 ±0.02 a	1.58 ±0.02 ab	1.59 ±0.02 a
Total	875.33 ±12.74 a	859.63 ±12.76 a	803.49 ±14.67 b	883.52 ±13.84 a	877.13 ±17.40 a	905.79 ±19.45 a	955.62 ±17.43 a	837.31 ±15.62 b	848.54 ±17.25 b	828.46 ±5.87 b
Antiradical acitvity	0.187 ±0.003 a	0.184 ±0.003 a	0.172 ±0.003 b	0.189 ±0.003 a	0.188 ±0.004 a	0.194 ±0.004 a	0.204 ±0.004 a	0.179 ±0.003 b	0.182 ±0.004 b	0.177 ±0.001 b

Values belonging to the same traits marked by different letters in the rows indicate significant differences between years, cultivars, or farming systems. Mean values for factors. ORG = organic, INT = integrated, CON = conventional, and MONO = monoculture farming system.

**Table 6:** Influence of the year, cultivar, and farming system on the content and antioxidant activity of alkylresorcinols. Mean alkylresorcinols (µg/g of the grain), total alkylresorcinol content (µg/g of the grain), and antiradical activity (in relation to caffeic acid’s activity = 1.00) of four winter wheat cultivars, cultivated in four farming system, over two years.

Alkyl- Resorcinol	Year		Cultivar				System			
	2017	2018	Fidelius	Hondia	Jantarka	KWS Ozon	ORG	CON	INT	MONO
C15:0	LOQ	LOQ	LOQ	LOQ	LOQ	LOQ	LOQ	LOQ	LOQ	LOQ
C17:0	27.77 ±1.18 a	27.68 ±0.81 a	32.17 ±1.15 a	27.46 ±0.75 b	20.32 ±1.03 c	30.96 ±1.30 a	30.34 ±1.77 a	27.11 ±1.42 b	25.99 ±1.12 b	27.47 ±1.21 ab
C19:1	LOQ	26.15 ± 1.70 a	15.99 ±3.50 a	13.21 ±3.61 b	13.17 ±2.82 b	9.93 ±2.74 c	6.01 ± 2.20 b	15.60 ±3.44 a	14.60 ± 3.16 a	16.10 ± 3.46 a
C21:2	42.24 ±1.20 a	36.65 ±1.15 b	34.48 ±1.26 b	39.81 ±1.49 a	41.89 ±1.90 a	41.60 ±1.92 a	42.38 ±1.67 a	38.87 ±2.08 ab	36.83 ±1.83 b	39.71 ±1.21 ab
C19:0	212.85 ±6.83 a	218.47 ±5.24 a	250.67 ±7.55 a	213.77 ±4.03 c	168.82 ±6.72 d	229.37 ±5.40 b	237.03 ±11.13 a	209.46 ±7.46 b	204.52 ±6.95 b	211.63 ±7.01 b
C21:1	LOQ	LOQ	LOQ	LOQ	LOQ	LOQ	LOQ	LOQ	LOQ	LOQ
C21:0	375.19 ±9.16 b	421.16 ±6.70 a	413.08 ±14.87 a	392.49 ±10.34 b	370.38 ±12.39 c	416.76 ±8.79 a	448.62 ±11.40 a	385.76 ±11.11 b	373.37 ±10.29 b	384.94 ±10.37 b
C23:0	79.27 ±2.18 b	95.44 ±1.96 a	94.20 ±4.35 a	82.08 ±2.93 c	83.74 ±3.07 bc	89.40 ±2.35 ab	99.30 ±3.98 a	85.76 ±2.69 b	81.17 ±2.70 b	83.19 ±2.72 b
C25:0	24.48 ±0.90 b	34.16 ±1.21 a	34.74 ±2.10 a	28.98 ±1.55 b	25.52 ±1.38 c	28.05 ±1.65 bc	34.29 ±1.67 a	27.21 ±1.23 b	24.11 ±1.23 b	31.68 ±2.23 a
Total	761.81 ±18.75 b	859.71 ±13.43 a	875.34 ±27.66 a	797.81 ±18.47 b	723.83 ±25.10 c	846.07 ±16.83 a	897.97 ±27.20 a	789.78 ±22.40 b	760.59 ±19.35 b	794.71 ±22.47 b
Antiradical acitvity	0.266 ±0.007 b	0.300 ±0.005 a	0.306 ±0.010 a	0.279 ±0.006 b	0.253 ±0.009 c	0.295 ±0.006 a	0.314 ±0.009 a	0.276 ±0.008 b	0.266 ±0.007 b	0.278 ±0.008 b

LOQ—below the limit of quantification. Values belonging to the same traits marked by different letters in the rows indicate significant differences between years, cultivars, or farming systems. ORG = organic, INT = integrated, CON = conventional, and MONO = monoculture farming system.

**Table 7:** Summary of analysis of variance results. Significance of effect (years, cultivars, and systems) in the analysis of variance and variability coefficient of alkylresorcinols.

Alkyl- Resorcinol	Source of Variability							V (%) ^#^
	Year (Y)	Cultivar (C)	System (S)	Y × C	Y × S	C × S	Y × C × S	
C15:0	ns	ns	ns	ns	ns	ns	ns	0.00
C17:0	ns	***	**	***	ns	ns	*	9.36
C19:1	***	***	***	***	***	***	***	7.22
C21:2	***	***	**	*	**	***	***	8.36
C19:0	ns	***	***	***	ns	***	***	8.07
C21:1	ns	ns	ns	ns	ns	ns	ns	0.00
C21:0	***	***	***	**	***	***	***	6.75
C23:0	***	***	***	ns	ns	***	**	10.30
C25:0	***	***	***	ns	***	ns	ns	8.38
Total	***	***	***	***	**	***	***	7.06
Antiradical acitvity	***	***	***	***	**	***	***	5.84

Significance levels are *** *p* < 0.001, ** *p* < 0.01; * *p* < 0.05, ns—not significant. ^#^ Variability coefficient V (%) = √S^2^/x × 100%.

## Data Availability

The original contributions presented in this study are included in the article/Supplementary Material. Further inquiries can be directed to the corresponding author.

## Supplementary Materials

The following supporting information can be downloaded at: https://www.mdpi.com/article/10.3390/molecules30040902/s1. Table S1. Yield (t/ha), ear planting (pcs*m^−2^), 1000 kernel weight (g) of winter wheat cultivated in an organic system. Table S2. Leaves infestation (% leaf area) of winter wheat cultivars by fungal pathogens in organic farming system. Table S3. Mean phenolic acids (µg/g of the grain ± SD), total phenolic acids content (µg/g of the grain ± SD) and antiradical activity (in relation to caffeic acid’s activity = 1.00) of winter wheat cultivars cultivated in organic farming system. Table S4. Summary of analysis of variance results. Significance of effect (years and cultivars) in the analysis of variance and variability coefficient of phenolic acids. Table S5. Mean alkylresorcinols (µg/g of the grain ± SD), total alkylresorcinols content (µg/g of the grain ± SD) and antiradical activity (in relation to α-tocopherol’s activity = 1.00) of winter wheat cultivars cultivated in the organic framing system. Table S6. Summary of analysis of variance results. Significance of effect (years and cultivars) in the analysis of variance and variability coefficient of alkylresorcinols. Table S7. Influence of cultivar for phenolic acid content (µg/g of the grain), in the study years (2017–2018). Table S8. Influence of farming system for phenolic acid content (µg/g of the grain), in the study years (2017–2018). Table S9. Phenolic acid content (µg/g of the grain) and antioxidant activity (in relation to caffeic acid’s activity = 1.00) of winter wheat cultivars. Table S10. Influence of cultivar for alkylresorcinols content (µg/g of the grain), in the study years (2017–2018). Table S11. Influence of farming system for alkylresorcinols content (µg/g of the grain), in the study years (2017–2018). Table S12. Alkylresorcinol content (µg/g of the grain) and antioxidant activity (in relation to caffeic acid’s activity = 1.00) of winter wheat cultivars. Table S13. Selected elements of the agricultural practice of winter wheat in different framing systems. Table S14. Calibration curve parameters for free phenolic acids reference standards. Figure S1. Effect of crop production systems on ears density of 4 winter wheat cultivars in 2-year study (A—2017, B—2018). *different letters in columns correspond to significant differences between means, according to Tukey’s test at *p* ≤ 0.05. Figure S2. Effect of crop production systems on thousand grain mass of 4 winter wheat cultivars in a 2-year study (A—2017, B—2018). * different letters in columns correspond to significant differences between means, according to Tukey’s test at *p* ≤ 0.05. Figure S3. Monthly air temperature and monthly of precipitation in Osiny for the period of 2016–2018.

## Abbreviations

Alkylresorcinols (ARs), 5-*n*-pentadecylresorcinol (C15:0), 5-*n*-heptadecylresorcinol (C17:0), 5-*n*-nonadecenylresorcinol (C19:1), 5-*n*-heneicosadienylresorcinol (C21:2), 5-*n*-nonadecylresorcinol (C19:0), 5-*n*-heneicosenylresorcinol (C21:1), 5-*n*-heneicosylresorcinol (C21:0), 5-*n*-tricosylresorcinol (C23:0), and 5-*n*-pentacosylresorcinol (C25:0), phenolic acids (PAs), hydrochloric acid (HCl), sodium hydroxide (NaOH), sodium hypochlorite (NaOCl), 2,2-diphenyl-1-picrylhydrazyl radical (DPPH^•^), thin-layer chromatography (TLC), ultra-performance liquid chromatography (UPLC), mass spectrometry (MS), photodiode array detector (PDA), analysis of variance (ANOVA), Biologische Bundesanstalt, Bundessortenamt und CHemische Industrie scale (BBCH scale), below the limit of quantification (LOQ), standard deviation (SD), cultivar (cv.), organic (ORG), conventional (CON), integrated (INT), monoculture (MONO).
