# Supplementary material for: The Effect of Farming Systems and Cultivars on the Qualitative and Quantitative Composition of Bioactive Compounds in Winter Wheat (Triticum aestivum L.)
Source: Molecules. 2025 Feb 15;30(4):902. doi: 10.3390/molecules30040902 (PMC11858240; doi:10.3390/molecules30040902)
Supplement: Supplementary file 1 [file molecules-30-00902-s001.zip › molecules-3462984-supplementary.pdf]

Table and Figure Contents:

Table S1. Yield (t/ha), ear planting (pcs*m^{-2}), 1000 kernel weight (g) of winter wheat cultivated in an organic system.

Table S2. Leaves infestation (% leaf area) of winter wheat cultivars by fungal pathogens in organic farming system.

Table S3. Mean phenolic acids (µg/g of the grain ± SD), total phenolic acids content (µg/g of the grain ± SD) and antiradical activity (in relation to caffeic acid's activity = 1.00) of winter wheat cultivars cultivated in organic farming system.

Table S4. Summary of analysis of variance results. Significance of effect (years and cultivars) in the analysis of variance and variability coefficient of phenolic acids.

Table S5. Mean alkylresorcinols (µg/g of the grain ± SD), total alkylresorcinols content (µg/g of the grain ± SD) and antiradical activity (in relation to α-tocopherol's activity = 1.00) of winter wheat cultivars cultivated in the organic framing system.

Table S6. Summary of analysis of variance results. Significance of effect (years and cultivars) in the analysis of variance and variability coefficient of alkylresorcinols.

Table S7. Influence of cultivar for phenolic acid content (µg/g of the grain), in the study years (2017-2018).

Table S8. Influence of farming system for phenolic acid content (µg/g of the grain), in the study years (2017-2018).

Table S9. Phenolic acid content (µg/g of the grain) and antioxidant activity (in relation to caffeic acid's activity = 1.00) of winter wheat cultivars.

Table S10. Influence of cultivar for alkylresorcinols content (µg/g of the grain), in the study years (2017-2018).

Table S11. Influence of farming system for alkylresorcinols content (µg/g of the grain), in the study years (2017-2018).

Table S12. Alkylresorcinol content (μg/g of the grain) and antioxidant activity (in relation to caffeic acid's activity = 1.00) of winter wheat cultivars.

Table S13. Selected elements of the agricultural practice of winter wheat in different framing systems.

Table S14. Calibration curve parameters for free phenolic acids reference standards.

Figure S1. Effect of crop production systems on ears density of 4 winter wheat cultivars in 2-year study (A- 2017, B – 2018). *different letters in columns correspond to significant differences between means, according to Tukey's test at $p \leq 0.05$.

Figure S2. Effect of crop production systems on thousand grain mass of 4 winter wheat cultivars in a 2-year study (A- 2017, B – 2018). * different letters in columns correspond to significant differences between means, according to Tukey's test at $p \leq 0.05$.

Figure S3. Monthly air temperature and monthly of precipitation in Osiny for the period of 2016–2018.

Table of Contents:

Table S1. Yield (t/ha), ear planting (pcs*m^{-2}), 1000 kernel weight (g) of winter wheat cultivated in an organic system.

Cultivar	Yield		Number of ears		1000 kernel weight	
	**2017**	**2018**	**2017**	**2018**	**2017**	**2018**
Arktis	3.12	2.99	346	325	36.7	33.2
Bellisa	4.08	3.37	324	314	44.2	35.7
Estivus	3.51	3.72	323	321	41.6	38.5
Fidelius	4.17	3.92	326	335	42.4	37.7
Hondia	3.97	3.89	338	303	44.9	44.7
Jantarka	3.95	3.97	356	333	43.5	42.3
KWS Ozon	3.00	3.30	341	308	40.8	38.7
Linus	3.64	2.78	288	312	37.6	38.6
Markiza	3.60	3.62	326	352	39.8	34.7
Ostka Strzelecka	3.22	3.20	312	299	40.8	35.1
Pokusa	3.38	2.87	293	329	38.2	33.9
Rokosz*	1.89	3.24	290	322	42.5	35.8
Mean	3.46	3.37	322	379	41.1	35.7
LSD α=0.05	0.33	0.49	93	56	0.95	1.6

*Yield and weight of one thousand grains of hulled grain, approximately 50% is husk.

Table S2. Leaves infestation (% leaf area) of winter wheat cultivars by fungal pathogens in organic farming system.

Cultivar	***Puccinia recondita***		***Septoria spp.***		***Drechslera tritici repentis***		***Puccinia striiformis***	
	**2017**	**2018**	**2017**	**2018**	**2017**	**2018**	**2017**	**2018**
Arktis	54.2d	2.8a	3.6ab	4.0a	1.9ab	8.8a	4.7bc	9.0a
Belissa	3.8a	6.5b	1.6a	5.0a	3.7a-c	8.3a	18.0f	9.0a
Estivus	12.2ab	5.8ab	2.3ab	6.0a	1.2a	8.3a	1.3ab	8.8a
Fidelius	21.9a-c	3.8ab	9.6b	2.75a	6.4c	8.8a	3.3a-c	9.0a
Hondia	20.9a-c	5.8ab	2.3ab	3.75a	5.2bc	7.8a	6.6cd	9.0a
Jantarka	34.0b-d	4.0ab	4.3ab	4.5a	3.7abc	8.3a	0a	8.8a
KWS Ozon	32.5b-d	3.5ab	4.9ab	5.75a	5.3bc	7.5a	0.2ab	9.0a
Linus	39.4cd	2.5a	1.07a	5.75a	0.5a	8.8a	0.3ab	8.8a
Markiza	44.0cd	4.8ab	3.7ab	3.5a	2.9a-c	8.3a	3.3a-c	8.8a
Ostka Strzelecka	51.5d	4.0ab	3.0ab	3.25a	1.2a	8.5a	11.1de	9.0a
Pokusa	38.5b-d	4.5ab	2.2ab	5.0a	2.8ab	8.0a	12.7e	9.0a
Rokosz	23.7a-c	6.8b	2.3ab	5.25a	2.6ab	7.8a	1.7ab	8.8a

Table S3. Mean phenolic acids (μg/g of the grain ± SD), total phenolic acids content (μg/g of the grain ± SD) and antiradical activity (in relation to caffeic acid's activity = 1.00) of winter wheat cultivars cultivated in organic farming system.

Cultivar	Year	Protocate-chuic acid	*p*-OH-Benzoic acid	Vanillic acid	Caffeic acid	Syringic acid	*p*-Coumaric acid	Ferulic acid	Sinapic acid	Salicylic acid	Total	Antiradical activity
Arktis	2017	5.90 ± 0.11b-g	2.02 ± 0.08f	24.08 ± 0.65c-g	37.90 ± 1.06c-e	10.80 ± 0.18j	33.73 ± 1.03c	794.20 ± 6.55b-f	41.74 ± 0.73e-j	1.50 ± 0.01h	951.87 ± 8.13d-i	0.204 ± 0.00e-i
	2018	5.25 ± 0.09e-i	2.39 ± 0.17ef	22.10 ± 0.84f-h	43.49 ± 1.10bc	11.47 ± 0.29h-j	38.40 ± 1.08c	796.49 ± 20.09b-f	42.04 ± 1.29e-i	1.74 ± 0.02cd	963.37 ± 22.56d-i	0.206 ± 0.00e-i
Belissa	2017	6.83 ± 0.26ab	5.49 ± 0.34bc	24.63 ± 0.14c-g	36.02 ± 1.91c-e	10.80 ± 0.18j	38.48 ± 5.89c	803.53 ± 16.96b-f	41.27 ± 1.03e-j	1.56 ± 0.03f-h	968.61 ± 24.00d-i	0.207 ± 0.01d-f
	2018	6.70 ± 0.13bc	5.24 ± 0.16cd	23.19 ± 0.31e-h	33.81 ± 1.34c-e	14.68 ± 0.10c-e	43.88 ± 6.05c	857.69 ± 8.71a-e	48.09 ± 0.81d-f	1.73 ± 0.03cd	1035.01 ± 12.42b-h	0.221 ± 0.00d-h
Estivus	2017	6.12 ± 0.16b-f	2.37 ± 0.14f	22.85 ± 0.75f-h	39.39 ± 1.78b-e	12.69 ± 0.48e-j	38.97 ± 4.68c	832.67 ± 16.40a-f	47.59 ± 0.75d-g	1.58 ± 0.01f-h	1004.23 ± 22.34c-i	0.215 ± 0.00d-i
	2018	4.94 ± 0.16g-i	3.20 ± 0.28e	22.65 ± 0.46f-h	33.08 ± 1.24c-e	13.84 ± 0.25d-h	36.15 ± 4.27c	883.62 ± 20.01a-d	49.59 ± 1.19c-e	1.69 ± 0.00c-e	1048.77 ± 28.91b-g	0.224 ± 0.01d-g
Fidelius	2017	6.27 ± 0.35b-d	2.05 ± 0.21f	24.93 ± 1.13c-g	30.47 ± 1.18c-e	15.73 ± 0.40cd	42.39 ± 2.99c	739.55 ± 40.46d-f	39.20 ± 1.12g-j	1.55 ± 0.01f-h	902.15 ± 46.81f-i	0.193 ± 0.01f-i
	2018	5.52 ± 0.23d-h	2.00 ± 0.11f	23.33 ± 0.62g-h	31.86 ± 1.16c-e	16.33 ± 0.51c	52.72 ± 7.91c	685.98 ± 19.59f	36.96 ± 1.01h-j	1.52 ± 0.01gh	856.23 ± 15.38hi	0.183 ± 0.00h
Hondia	2017	4.96 ± 0.16g-i	2.03 ± 0.15f	22.62 ± 0.66f-h	25.76 ± 1.18e	16.00 ± 0.72cd	37.72 ± 3.49c	770.38 ± 31.98c-f	39.65 ± 1.38f-j	1.56 ± 0.02f-h	920.68 ± 37.72e-i	0.197 ± 0.01e-i
	2018	4.47 ± 0.07i	2.27 ± 0.06ef	20.87 ± 0.26gh	27.90 ± 0.46de	15.82 ± 0.31cd	30.83 ± 0.68c	744.22 ± 17.94d-f	39.36 ± 1.11g-j	1.50 ± 0.00gh	887.24 ± 20.16g-i	0.190 ± 0.00g-i
Jantarka	2017	5.39 ± 0.14d-i	1.97 ± 0.08f	21.57 ± 0.21gh	38.90 ± 0.38c-e	11.47 ± 0.04h-j	41.67 ± 2.59c	842.44 ± 31.94a-e	57.43 ± 2.06bc	1.58 ± 0.01f-h	1022.43 ± 36.77b-i	0.219 ± 0.01b-i
	2018	4.44 ± 0.02i	2.24 ± 0.14ef	19.30 ± 0.19h	36.55 ± 0.37c-e	11.34 ± 0.08ij	33.34 ± 2.10c	807.02 ± 7.63b-f	57.38 ± 1.49bc	1.51 ± 0.01gh	973.13 ± 10.46d-i	0.208 ± 0.00f-i
KWS Ozon	2017	5.63 ± 0.13d-h	2.65 ± 0.08ef	26.06 ± 0.51c-f	59.99 ± 11.34a	13.60 ± 0.36d-i	48.20 ± 0.73c	808.64 ± 2.69b-f	42.72 ± 1.11d-i	1.74 ± 0.04cd	1009.23 ± 15.44b-i	0.216 ± 0.00b-i

	2018	5.01 ± 0.28g-i	2.81 ± 0.16ef	23.40 ± 0.72g-h	29.95 ± 1.98c-e	14.38 ± 0.26c-f	52.46 ± 9.55c	893.38 ± 31.33a-c	50.86 ± 1.47cd	1.59 ± 0.02e-h	1073.83 ± 43.30b-f	0.230 ± 0.01d-f
Linus	2017	5.19 ± 0.05f-i	3.17 ± 0.26e	22.83 ± 0.98f-h	38.27 ± 2.41c-e	14.13 ± 0.38c-g	48.12 ± 0.12c	892.84 ± 46.40a-c	60.42 ± 4.13b	1.72 ± 0.01cd	1086.70 ± 50.03b-e	0.232 ± 0.01c-e
	2018	5.10 ± 0.03g-i	4.31 ± 0.06d	27.18 ± 1.70c-e	43.47 ± 3.29bc	15.73 ± 0.29cd	48.89 ± 1.45c	966.44 ± 7.29a	70.53 ± 2.14a	1.77 ± 0.00bc	1183.43 ± 16.26bc	0.253 ± 0.00c
Markiza	2017	5.61 ± 0.26d-h	4.34 ± 0.36d	27.60 ± 0.99cd	33.03 ± 1.86c-e	26.68 ± 1.19a	37.01 ± 1.99c	938.02 ± 45.40ab	46.97 ± 1.85d-g	1.60 ± 0.01e-g	1120.87 ± 53.54b-d	0.240 ± 0.01cd
	2018	5.45 ± 0.16d-h	4.76 ± 0.10cd	28.08 ± 0.21c	33.17 ± 1.11c-e	26.77 ± 0.56a	46.24 ± 4.59c	872.78 ± 19.70a-e	39.31 ± 1.06g-j	1.75 ± 0.01c	1058.33 ± 25.13b-g	0.226 ± 0.01d-g
Ostka Strzelecka	2017	6.20 ± 0.31b-e	2.41 ± 0.30ef	24.28 ± 1.34c-g	42.14 ± 2.92b-d	12.03 ± 0.40f-j	53.01 ± 6.43c	870.76 ± 46.68a-e	40.66 ± 1.99f-j	1.64 ± 0.03d-f	1053.12 ± 59.61b-g	0.225 ± 0.01e-g
	2018	5.16 ± 0.13f-i	2.36 ± 0.10ef	23.75 ± 0.30d-g	32.98 ± 0.29c-e	12.22 ± 0.28f-j	45.85 ± 2.37c	874.09 ± 25.93a-e	38.35 ± 1.38h-j	1.76 ± 0.04c	1036.52 ± 29.76b-g	0.222 ± 0.01c-g
Pokusa	2017	4.84 ± 0.02hi	1.85 ± 0.09f	22.59 ± 0.20f-h	30.51 ± 0.12c-e	11.66 ± 0.28h-j	51.16 ± 0.29c	728.82 ± 13.00ef	44.63 ± 0.86d-h	1.59 ± 0.00e-h	897.65 ± 13.89f-i	0.192 ± 0.00g-i
	2018	4.45 ± 0.17i	2.31 ± 0.22ef	21.50 ± 0.94gh	28.03 ± 1.09de	11.86 ± 0.63g-j	45.79 ± 2.86c	691.96 ± 40.36f	43.17 ± 1.96d-i	1.76 ± 0.04c	850.83 ± 44.30i	0.182 ± 0.01i
Rokosz	2017	7.72 ± 0.12a	7.73 ± 0.08a	48.25 ± 1.35a	54.18 ± 0.92ab	18.96 ± 0.41b	319.98 ± 14.25a	926.20 ± 12.30ab	32.99 ± 0.58i	1.98 ± 0.01a	1417.97 ± 27.97a	0.303 ± 0.01a
	2018	5.75 ± 0.18c-h	6.32 ± 0.17b	37.11 ± 0.68b	33.51 ± 1.26c-e	15.34 ± 0.56cd	209.90 ± 10.76b	841.73 ± 31.68a-e	35.19 ± 0.94ij	1.87 ± 0.04ab	1186.73 ± 45.87b	0.254 ± 0.01b

Values are expressed as mean ±SD. Means in a column followed by different letters show significant differences ($p < 0.05$) according to the Tukey test.

Table S4. Summary of analysis of variance results. Significance of effect (years and cultivars) in the analysis of variance and variability coefficient of phenolic acids.

**Phenolic acid**	**Source of variability**			**V (%)[#]**
	**Year (Y)**	**Cultivar (C)**	**Y*C**	
Protocatechuic acid	***	***	***	5.54

p-OH-Benzoic acid	*	***	***	9.82
Vanillic acid	***	***	***	5.44
Caffeic acid	***	***	***	9.12
Syringic acid	*	***	***	5.22
p-Coumaric acid	***	***	***	8.25
Ferulic acid	ns	***	*	5.65
Sinapic acid	*	***	***	6.01
Salicylic acid	***	***	***	1.99
Total	ns	***	**	5.61
Antiradical acitvity	ns	***	**	5.49

Significance levels are *** $P < 0.001$, ** $P < 0.01$; * $P < 0.05$, ns – not significant;
[#] Variability coefficient V (%) = $\sqrt{S^2}/x*100\%$

Table S5. Mean alkylresorcinols (μg/g of the grain ± SD), total alkylresorcinols content (μg/g of the grain ± SD) and antiradical activity (in relation to α-tocopherol's activity = 1.00) of winter wheat cultivars cultivated in the organic framing system.

**Cultivar**	**Year**	**C15:0**	**C17:0**	**C19:1**	**C21:2**	**C19:0**	**C21:1**	**C21:0**	**C23:0**	**C25:0**	**Total**	**Antiradical activity**
Arktis	2017	LOQ	24.10 ± 0.20a-d	LOQ	23.11 ± 0.35fg	198.45 ± 13.19d-h	LOQ	421.80 ± 1.82b-g	93.18 ± 0.59bc	39.59 ± 13.27ab	800.24 ± 9.54c-g	0.282 ± 0.00cd
	2018	LOQ	20.67 ± 3.26a-d	21.70 ± 3.05d	21.89 ± 3.49g	204.40 ± 4.18d-g	LOQ	420.58 ± 3.52b-h	96.87 ± 0.71bc	34.55 ± 1.25ab	820.66 ± 4.83b-g	0.289 ± 0.00b-d
Belissa	2017	LOQ	33.40 ± 4.39a-c	LOQ	34.64 ± 3.65b-g	284.92 ± 8.03ab	LOQ	473.18 ± 14.03b	89.09 ± 11.34bc	41.94 ± 5.53ab	957.17 ± 35.17b	0.337 ± 0.01b

	2018	LOQ	32.44 ± 0.76a-d	37.51 ± 0.56a	35.42 ± 1.16b-g	253.29 ± 3.09bc	LOQ	446.33 ± 4.60b-d	100.97 ± 1.27bc	51.87 ± 1.17a	957.85 ± 10.83b	0.336 ± 0.00b
Estivus	2017	LOQ	24.95 ± 0.68b-d	LOQ	47.96 ± 0.63a-d	186.70 ± 0.52f-i	LOQ	406.64 ± 0.99c-h	87.19 ± 0.38bc	23.42 ± 0.48b	776.86 ± 1.17c-g	0.274 ± 0.00cd
	2018	LOQ	26.74 ± 1.16a-d	27.79 ± 1.20cd	45.48 ± 1.68a-e	207.16 ± 7.42d-g	LOQ	462.80 ± 17.13bc	101.90 ± 4.15bc	34.58 ± 1.87ab	906.46 ± 34.52bc	0.319 ± 0.01bc
Fidelius	2017	LOQ	37.65 ± 0.57ab	LOQ	42.96 ± 0.66a-e	276.64 ± 4.36b	LOQ	467.80 ± 9.13bc	106.69 ± 2.81ab	36.35 ± 1.39ab	968.10 ± 18.63b	0.341 ± 0.01b
	2018	LOQ	34.98 ± 7.58ab	22.21 ± 1.87d	29.27 ± 5.82e-g	320.71 ± 14.44a	LOQ	547.20 ± 25.58a	138.78 ± 7.62a	44.96 ± 9.10ab	1138.12 ± 58.48a	0.401 ± 0.02a
Hondia	2017	LOQ	27.64 ± 2.35a-d	LOQ	42.12 ± 7.79a-e	229.10 ± 14.73cd	LOQ	472.67 ± 11.33b	82.18 ± 14.88bc	29.28 ± 6.12b	882.98 ± 45.08b-d	0.311 ± 0.02b-d
	2018	LOQ	29.38 ± 1.03a-d	LOQ	40.19 ± 1.76a-e	228.55 ± 3.76cd	LOQ	433.27 ± 16.17b-e	97.46 ± 4.50bc	36.66 ± 1.69ab	865.50 ± 28.29b-e	0.305 ± 0.01b-e
Jantarka	2017	LOQ	19.66 ± 1.21a-d	LOQ	49.06 ± 0.50a-c	162.61 ± 6.61hi	LOQ	391.07 ± 15.93d-i	85.45 ± 4.23bc	25.06 ± 1.85b	732.91 ± 30.14e-g	0.258 ± 0.01e-f
	2018	LOQ	19.04 ± 0.62a-d	25.86 ± 1.82cd	42.95 ± 1.35a-e	159.91 ± 2.09i	LOQ	379.29 ± 6.31e-i	92.26 ± 1.55bc	36.23 ± 0.15ab	755.53 ± 11.48d-g	0.266 ± 0.00d-g
KWS Ozon	2017	LOQ	39.28 ± 2.49a	LOQ	50.94 ± 2.79ab	268.91 ± 13.19b	LOQ	469.76 ± 22.20bc	99.09 ± 4.78bc	31.65 ± 1.74ab	959.63 ± 47.12b	0.338 ± 0.02b
	2018	LOQ	35.12 ± 1.56ab	LOQ	41.55 ± 0.90a-e	249.78 ± 6.03bc	LOQ	427.93 ± 10.17b-f	92.49 ± 2.28bc	34.11 ± 1.41ab	880.98 ± 21.77b-e	0.310 ± 0.01b-d
Linus	2017	LOQ	30.84 ± 6.79a-d	LOQ	34.17 ± 7.81c-g	278.59 ± 3.57b	LOQ	366.45 ± 13.87f-i	87.08 ± 17.14bc	29.31 ± 6.63b	826.44 ± 39.71b-g	0.291 ± 0.01b-g
	2018	LOQ	29.99 ± 0.48a-d	34.65 ± 1.83ab	32.10 ± 0.17d-g	219.44 ± 0.82c-f	LOQ	390.00 ± 1.80d-i	89.54 ± 1.66bc	33.33 ± 0.49ab	829.06 ± 5.23b-g	0.292 ± 0.00b-d
Markiza	2017	LOQ	25.50 ± 0.83a-d	LOQ	53.07 ± 1.71a	181.45 ± 1.76g-i	LOQ	339.84 ± 3.67i	78.72 ± 1.13bc	32.39 ± 0.30ab	710.97 ± 8.83fg	0.250 ± 0.01f
	2018	LOQ	24.29 ± 0.66a-d	LOQ	44.81 ± 1.84a-e	190.13 ± 2.97e-i	LOQ	359.67 ± 6.59g-i	88.48 ± 1.16bc	42.29 ± 0.31ab	749.66 ± 12.50d-g	0.264 ± 0.00d-f
Ostka Strzelecka	2017	LOQ	24.87 ± 0.31b-d	LOQ	43.48 ± 1.16a-e	198.96 ± 1.74d-h	LOQ	354.84 ± 3.31hi	83.60 ± 1.05bc	35.08 ± 0.19ab	740.82 ± 4.02d-g	0.261 ± 0.00d-g
	2018	LOQ	27.10 ± 1.85a-d	29.97 ± 2.58bc	39.80 ± 2.84a-f	225.37 ± 8.31c-e	LOQ	390.52 ± 14.63d-i	90.77 ± 5.77bc	43.27 ± 2.76ab	846.79 ± 37.38b-f	0.298 ± 0.01c-e

Pokusa	2017	LOQ	27.87 ± 0.31a-d	LOQ	49.09 ± 0.68a-c	194.33 ± 3.10d-i	LOQ	336.51 ± 5.66i	72.26 ± 1.59c	LOQ	680.06 ± 11.60g	0.240 ± 0.00g
	2018	LOQ	28.70 ± 0.40a-d	40.62 ± 1.29a	40.15 ± 1.36a-e	209.27 ± 3.01d-g	LOQ	369.31 ± 7.35e-i	82.27 ± 2.16bc	33.55 ± 0.33ab	803.87 ± 15.37c-g	0.283 ± 0.01c-g
Rokosz	2017	LOQ	23.98 ± 0.97a-d	LOQ	40.22 ± 2.38a-e	197.03 ± 9.93d-h	LOQ	369.36 ± 18.67e-i	81.03 ± 3.52bc	LOQ	711.62 ± 35.31fg	0.251 ± 0.01fg
	2018	LOQ	28.98 ± 0.43a-d	30.43 ± 1.98bc	36.82 ± 1.53a-g	227.13 ± 4.02cd	LOQ	428.62 ± 7.70b-f	91.68 ± 0.72bc	LOQ	843.67 ± 13.33b-f	0.297 ± 0.00c-e

* - different letter within a column indicates significant differences ($p < 0.05$),
LOQ-below the limit of quantification.

Table S6. Summary of analysis of variance results. Significance of effect (years and cultivars) in the analysis of variance and variability coefficient of alkylresorcinols.

Alkyl-resorcinol	Source of variability			V (%)[#]
	Year (Y)	Cultivar (C)	Y*C	
C15:0	ns	ns	ns	0.00
C17:0	ns	***	ns	8.84
C19:1	***	***	***	8.12
C21:2	***	***	ns	7.28
C19:0	ns	***	***	5.25
C21:1	ns	ns	ns	0.00
C21:0	***	***	***	5.07
C23:0	***	***	ns	8.24
C25:0	***	***	*	9.59
Total	***	***	***	5.66
Antiradical acitvity	***	***	***	5.75

Significance levels are *** $P < 0.001$, ** $P < 0.01$; * $P < 0.05$, ns – not significant;
[#] Variability coefficient V (%) = $\sqrt{S^2}/x*100\%$

Table S7. Influence of cultivar for phenolic acid content (µg/g of the grain), in the study years (2017-2018).

Phenolic acid	2017				2018			
	Fidelius	Hondia	Jantarka	KWS Ozon	Fidelius	Hondia	Jantarka	KWS Ozon
CAF	21.13 ± 1.73c	20.40 ± 1.23c	26.58 ± 2.19b	28.89 ± 5.93a	22.29 ± 1.80a	21.44 ± 1.16a	26.15 ± 1.92a	21.17 ± 1.60a
SYR	16.67 ± 0.31b	17.88 ± 0.45a	12.43 ± 0.27d	14.38 ± 0.23c	16.81 ± 0.31b	17.23 ± 0.37a	10.99 ± 0.14d	14.06 ± 0.26c
PCO	28.56 ± 2.57b	43.59 ± 2.60a	37.99 ± 1.81a	33.17 ± 3.08ab	25.95 ± 4.99a	24.97 ± 1.36a	23.73 ± 1.99a	30.22 ± 4.41a
SIN	35.26 ± 0.94d	41.69 ± 0.91c	53.44 ± 1.27a	44.06 ± 0.90b	34.64 ± 0.62d	42.32 ± 1.30c	52.08 ± 1.60a	47.99 ± 1.33b
SAL	1.49 ± 0.01b	1.55 ± 0.01a	1.51 ± 0.01b	1.57 ± 0.03a	1.62 ± 0.02b	1.64 ± 0.03b	1.63 ± 0.02b	1.67 ± 0.02a

Comparison of averages for combinations of years (Y) * cultivars (C) – C/Y. Values are expressed as mean ±SD. Means in a row followed by different letters show significant differences ($p < 0.05$) according to the Tukey test.

Table S8. Influence of farming system for phenolic acid content (µg/g of the grain), in the study years (2017-2018).

Phenolic acid	2017				2018			
	ORG	CON	INT	MONO	ORG	CON	INT	MONO
PRO	5.56 ± 0.17a	4.04 ± 0.08bc	4.42 ± 0.10b	3.95 ± 0.11c	4.86 ± 0.15a	3.45 ± 0.09b	3.55 ± 0.09b	3.79 ± 0.12b
POH	2.17 ± 0.10a	1.73 ± 0.11b	1.76 ± 0.15b	1.87 ± 0.10b	2.33 ± 0.10a	1.44 ± 0.15b	1.70 ± 0.17b	2.22 ± 0.05a
VAN	23.80 ± 0.62a	21.75 ± 0.45b	21.39 ± 0.55b	21.71 ± 0.89b	21.72 ± 0.56a	20.90 ± 0.64a	20.30 ± 0.55a	21.76 ± 0.71a
CAF	38.78 ± 4.65a	20.92 ± 0.46b	19.54 ± 0.93b	17.76 ± 0.71b	31.57 ± 1.09a	18.39 ± 0.42b	19.92 ± 0.78b	21.17 ± 0.75b
SYR	14.20 ± 0.58b	15.80 ± 0.63a	15.85 ± 0.74a	15.51 ± 0.77a	14.47 ± 0.60a	15.41 ± 0.95a	14.20 ± 0.78a	15.01 ± 0.80a
PCO	42.50 ± 1.60a	34.15 ± 1.87b	30.85 ± 2.68b	35.81 ± 4.20b	42.34 ± 4.10a	18.42 ± 1.00b	21.39 ± 1.10b	22.73 ± 0.97b
SIN	44.75 ± 2.33a	41.13 ± 2.06a	46.27 ± 2.43a	42.29 ± 1.60a	46.14 ± 2.58a	44.67 ± 2.21b	46.17 ± 2.59a	40.04 ± 1.22c
SAL	1.61 ± 0.02a	1.50 ± 0.01b	1.50 ± 0.01b	1.51 ± 0.01b	1.53 ± 0.01b	1.69 ± 0.01a	1.67 ± 0.01a	1.68 ± 0.01a

Comparison of averages for combinations of years (Y) * systems (S) – S/Y. Values are expressed as mean ±SD. Means in a row followed by different letters show significant differences ($p < 0.05$) according to the Tukey test.
ORG=organic, INT=integrated, CON=conventional and MONO=monoculture farming system.

Table S9. Phenolic acid content (µg/g of the grain) and antioxidant activity (in relation to caffeic acid's activity = 1.00) of winter wheat cultivars.

Phenolic acid	Fidelius				Hondia			
	ORG	CON	INT	MONO	ORG	CON	INT	MONO
PRO	5.90 ± 0.25a	4.05 ± 0.11c	4.12 ± 0.15c	4.51 ± 0.05b	4.71 ± 0.13a	3.86 ± 0.19bc	4.22 ± 0.28b	3.75 ± 0.07c

POH	2.02 ± 0.11a	1.19 ± 0.14b	1.05 ± 0.09b	2.06 ± 0.11a	2.15 ± 0.09a	1.93 ± 0.13a	2.04 ± 0.14a	1.84 ± 0.14a
VAN	24.13 ± 0.68b	22.73 ± 0.22b	22.79 ± 0.39b	25.64 ± 0.52a	21.75 ± 0.50a	22.57 ± 0.51a	21.78 ± 0.61a	22.09 ± 0.51a
CAF	31.16 ± 0.80a	18.74 ± 0.99b	17.39 ± 0.57b	19.57 ± 1.26b	26.83 ± 0.61a	20.22 ± 0.79b	19.46 ± 0.58b	17.16 ± 1.07b
SYR	16.03 ± 0.32b	16.90 ± 0.27b	16.14 ± 0.41b	17.89 ± 0.25a	15.91 ± 0.35d	18.68 ± 0.32a	18.11 ± 0.65b	17.51 ± 0.26c
PCO	47.56 ± 4.43a	20.39 ± 2.59b	19.79 ± 1.71b	21.29 ± 0.83b	34.28 ± 2.22b	30.05 ± 4.68b	32.72 ± 4.75b	40.07 ± 6.85a
FER	712.77 ± 23.40a	632.60 ± 10.02b	626.98 ± 19.16b	712.54 ± 4.99a	757.30 ± 17.41a	761.98 ± 21.54a	761.69 ± 27.97a	675.03 ± 11.94b
SIN	38.08 ± 0.84a	32.25 ± 0.60c	33.96 ± 0.88b	35.49 ± 0.40b	39.50 ± 0.80b	43.12 ± 1.52b	45.63± 1.44a	39.77 ± 1.04b
SAL	1.54 ± 0.01a	1.58 ± 0.04a	1.55 ± 0.04a	1.57 ± 0.04a	1.53 ± 0.02b	1.63 ± 0.04a	1.62 ± 0.03a	1.61 ± 0.03a
Total	879.19 ± 24.31a	750.42 ± 9.68c	743.78 ± 20.86c	840.57 ± 5.89b	903.96 ± 20.54a	904.03 ± 24.41a	907.27 ± 34.40a	818.83 ± 14.30b
Antiradical acitvity	0.188 ± 0.005a	0.161 ± 0.002c	0.159 ± 0.004c	0.180 ± 0.001b	0.193 ± 0.004a	0.193 ± 0.005a	0.194 ± 0.007a	0.175 ± 0.003b

**Phenolic acid**	**Jantarka**				**KWS Ozon**			
	**ORG**	**CON**	**INT**	**MONO**	**ORG**	**CON**	**INT**	**MONO**
PRO	4.92 ± 0.22a	3.54 ± 0.19b	3.82 ± 0.20b	3.68 ± 0.08b	5.32 ± 0.20a	3.53 ± 0.10b	3.77 ± 0.27b	3.53 ± 0.05b
POH	2.11 ± 0.10a	1.22 ± 0.06c	1.60 ± 0.16bc	1.91 ± 0.11b	2.73 ± 0.09a	2.00 ± 0.13c	2.23 ± 0.05c	2.38 ± 0.07b
VAN	20.44 ± 0.52a	18.59 ± 0.51b	18.55 ± 0.46b	19.20 ± 0.16b	24.73 ± 0.71a	21.40 ± 0.28b	20.27 ± 0.31b	20.00 ± 0.36b
CAF	37.72 ± 0.58a	20.94 ± 0.69b	23.95 ± 0.77b	22.83 ± 0.79b	44.97 ± 8.46a	18.73± 0.42b	18.13 ± 0.25b	18.29 ± 0.27b
SYR	11.40 ± 0.05a	11.95 ± 0.60a	11.78 ± 0.65a	11.69 ± 0.22a	13.99 ± 0.27a	14.90 ± 0.43a	14.07 ± 0.38a	13.94 ± 0.20a
PCO	37.51 ± 2.39a	25.90 ± 4.28b	28.47 ± 2.87b	31.57 ± 5.03b	50.33 ± 4.39a	28.80 ± 3.36b	23.50 ± 1.36b	24.14 ± 0.90b
FER	824.73 ± 16.69a	679.41 ± 10.84b	717.89 ± 19.44b	693.86 ± 9.13b	851.01 ± 23.60a	744.46 ± 22.36b	746.64 ± 8.13b	694.82 ± 11.70b
SIN	57.40 ± 1.14a	50.89 ± 0.66c	55.54 ± 1.46b	47.20 ± 1.46d	46.79 ± 1.20b	45.35 ± 1.69c	49.75 ± 0.71a	42.20 ± 1.08c
SAL	1.55 ± 0.02b	1.57 ± 0.05b	1.57 ± 0.04b	1.60 ± 0.04a	1.66 ± 0.04a	1.62 ± 0.04b	1.60 ± 0.04b	1.59 ± 0.04b
Total	997.78 ± 20.34a	814.01 ± 16.03b	863.17 ± 25.17b	833.54 ± 11.86b	1041.53 ± 25.13a	880.79 ± 25.65b	879.95 ± 9.79b	820.89 ± 13.78b
Antiradical acitvity	0.213 ± 0.004a	0.174 ± 0.003b	0.185 ± 0.005b	0.178 ± 0.003b	0.223 ± 0.005a	0.188 ± 0.005b	0.188 ± 0.002b	0.176 ± 0.003b

Comparison of averages for combinations of cultivars (C) * systems (S) – C/Y. Values are expressed as mean ±SD. Means in a row followed by different letters show significant differences ($p < 0.05$) according to the Tukey test.
ORG=organic, INT=integrated, CON=conventional and MONO=monoculture farming system.

Table S10. Influence of cultivar for alkylresorcinols content (µg/g of the grain), in the study years (2017-2018).

**Alkyl-resorcinol**	**2017**				**2018**			
	**Fidelius**	**Hondia**	**Jantarka**	**KWS Ozon**	**Fidelius**	**Hondia**	**Jantarka**	**KWS Ozon**
C17:0	33.68 ± 1.38a	25.66 ± 1.29b	17.25± 0.91b	34.51 ± 1.25a	30.66 ± 1.80a	29.26 ± 0.31a	23.39 ± 1.37a	27.41 ± 1.79a

C19:1	LOQ	LOQ	LOQ	LOQ	31.98 ± 2.16a	26.43 ± 4.77b	26.33 ± 1.33b	19.87 ± 3.69c
C21:2	34.38 ± 1.86b	42.30 ± 2.61a	46.26 ± 1.63a	46.03 ± 1.81a	34.58 ± 1.82b	37.32 ± 1.15a	37.53 ± 2.98a	37.18 ± 2.92a
C19:0	257.19 ± 6.23a	206.45 ± 7.13c	148.17 ± 4.87d	239.60 ± 7.56b	244.14 ± 13.86a	221.10 ± 2.67a	189.48 ± 9.36b	219.14 ± 6.76a
C21:0	392.38 ± 19.52a	379.68 ± 18.31a	328.04 ± 14.15b	400.65 ± 15.14a	433.78 ± 21.59a	405.30 ± 9.06a	412.71 ± 10.77a	432.86 ± 6.83a
Total	834.41 ± 33.62a	751.62 ± 30.30b	632.05 ± 25.30c	829.16 ± 29.81a	916.27 ± 42.01a	843.99 ± 10.97b	815.61 ± 21.54b	862.98 ± 15.64b
Antiradical acitvity	0.291 ± 0.012a	0.262 ± 0.011b	0.221 ± 0.009c	0.290 ± 0.010a	0.320 ± 0.015a	0.295 ± 0.004b	0.285 ± 0.008b	0.301 ± 0.005b

Comparison of averages for combinations of years (Y) * cultivars (C) – C/Y. Values are expressed as mean ±SD. Means in a row followed by different letters show significant differences ($p < 0.05$) according to the Tukey test.

Table S11. Influence of farming system for alkylresorcinols content (μg/g of the grain), in the study years (2017-2018).

Alkyl-resorcinol	2017				2018			
	**ORG**	**CON**	**INT**	**MONO**	**ORG**	**CON**	**INT**	**MONO**
C19:1	LOQ	LOQ	LOQ	LOQ	12.02 ± 3.69b	31.19 ± 2.31a	29.19 ± 1.75a	32.20 ± 1.69a
C21:2	46.27 ± 2.11a	42.84 ± 2.71a	41.01 ± 2.38a	38.85 ± 2.11a	38.49 ± 2.11b	34.91 ± 2.82b	32.65 ± 2.28b	40.56 ± 1.25a
C21:0	450.32 ± 12.26a	360.88 ± 16.58b	332.92 ± 11.11b	356.63 ± 12.71b	446.92 ± 19.83a	410.67 ± 11.36a	413.81 ± 4.69a	413.25 ± 11.92a
C25:0	30.59 ± 1.90a	24.67 ± 1.62b	20.20 ± 0.84b	22.47 ± 1.27b	37.99 ± 2.35a	29.74 ± 1.68b	28.02 ± 1.68b	40.88 ± 1.93a
Total	885.91 ± 32.55a	743.81 ± 39.03b	689.25 ± 20.87b	728.28 ± 31.30b	910.03 ± 44.81a	835.75 ± 13.79a	831.93 ± 14.31a	861.13 ± 18.14a
Antiradical acitvity	0.309 ± 0.011a	0.260 ± 0.014b	0.241 ± 0.007b	0.254 ± 0.011b	0.318 ± 0.016a	0.292 ± 0.005a	0.291 ± 0.005a	0.301 ± 0.006a

Comparison of averages for combinations of years (Y) * systems (S) – S/Y. Values are expressed as mean ± SD. Means in a row followed by different letters show significant differences ($p < 0.05$) according to the Tukey test. ORG=organic, INT=integrated, CON=conventional and MONO=monoculture farming system.

Table S12. Alkylresorcinol content (μg/g of the grain) and antioxidant activity (in relation to caffeic acid's activity = 1.00) of winter wheat cultivars.

Alkyl-resorcinol	Fidelius				Hondia			
	**ORG**	**CON**	**INT**	**MONO**	**ORG**	**CON**	**INT**	**MONO**
C19:1	11.12 ± 5.04c	20.31 ± 9.11a	17.33 ± 7.79ab	15.22 ± 6.86b	LOQ	17.33 ± 7.79b	15.21 ± 6.86b	20.31 ± 9.11a
C21:2	36.12 ± 4.03a	36.58 ± 1.97a	31.08 ± 2.04a	34.15 ± 1.38a	41.16 ± 3.60a	34.64 ± 1.78b	41.43 ± 3.60a	42.00 ± 3.14a
C19:0	298.68 ± 11.94a	233.58 ± 13.83b	231.42 ± 7.35b	238.99 ± 6.39b	228.83 ± 6.80a	201.32 ± 10.41a	214.39 ± 7.91a	210.56 ± 1.62a

C21:0	507.50 ± 21.51a	387.50 ± 15.53b	353.83 ± 23.95c	403.50 ± 12.56b	452.97± 12.47a	352.77 ± 20.01b	396.23 ± 15.15b	367.97 ± 3.57b
C23:0	122.73 ± 8.04a	87.40 ± 3.90b	79.53 ± 6.12b	87.15 ± 2.33b	89.82 ± 7.75a	78.63 ± 5.75a	82.63 ± 4.42a	77.26 ± 5.14a
Total	1053.11 ± 46.89a	829.04 ± 30.79b	770.61 ± 36.43b	848.59 ± 22.80b	874.24 ± 24.09a	739.32 ± 46.65b	803.47 ± 33.77ab	774.19 ± 20.17b
Antiradical acitvity	0.368 ± 0.016a	0.290 ± 0.011b	0.269 ± 0.013b	0.296 ± 0.008b	0.305 ± 0.008a	0.258 ± 0.016b	0.281 ± 0.012b	0.270 ± 0.007b
**Alkyl-resorcinol**	**Jantarka**				**KWS Ozon**			
	**ORG**	**CON**	**INT**	**MONO**	**ORG**	**CON**	**INT**	**MONO**
C19:1	12.93 ± 5.84b	13.90 ± 6.24b	10.85 ± 5.04b	14.98 ± 6.80a	LOQ	10.85 ± 5.04b	14.98 ± 6.80a	13.90 ± 6.24b
C21:2	46.00 ± 1.51a	47.54 ± 2.41a	33.25 ± 5.45b	40.78 ± 1.84a	46.25 ± 2.47a	36.73 ± 6.82a	41.57 ± 1.57a	41.88 ± 1.88a
C19:0	161.26 ± 13.16b	176.90 ± 15.15a	161.35 ± 13.16b	175.78 ± 19.32a	259.34 ± 7.77a	226.05 ± 9.91b	210.93 ± 7.26c	221.17 ± 7.83b
C21:0	385.17 ± 8.10a	390.71 ± 35.37a	363.64 ± 24.61b	341.97 ± 24.62b	448.84 ± 14.38a	412.11 ± 4.35bc	379.76 ± 18.21c	426.31 ± 19.03b
C23:0	88.85 ± 2.52a	86.30 ± 7.81a	82.38 ± 6.74a	77.42 ± 6.77a	95.78 ± 2.79a	90.71 ± 2.79a	80.14 ± 5.46ab	90.94 ± 5.46a
Total	774.20 ± 15.29a	760.31 ± 68.61a	692.78 ± 44.41b	698.03 ± 63.31b	920.31 ± 29.12a	830.45 ± 8.68bc	775.49 ± 33.12c	858.01 ± 31.92b
Antiradical acitvity	0.260 ± 0.005ab	0.266 ± 0.024a	0.242 ± 0.016b	0.244 ± 0.022b	0.321 ± 0.010a	0.290 ± 0.003b	0.271 ± 0.011b	0.300 ± 0.011b

Comparison of averages for combinations of cultivars (C) * systems (S) – S/C. Values are expressed as mean ±SD. Means in a row followed by different letters show significant differences ($p < 0.05$) according to the Tukey test. LOQ-below the limit of quantification.
ORG=organic, INT=integrated, CON=conventional and MONO=monoculture farming system.

Table S13. Selected elements of the agricultural practice of winter wheat in different framing systems.

**Specification**	**Farming system**			
	**Organic (ORG)**	**Conventional (CON)**	**Integrated (INT)**	**Monoculture (MONO)**
Crop rotation	Z^{++}- P.j. - K.c. - P.o. * - O+V	Rz. - P.o. - P.j. *	Z^{++}- P.j. - K.c. - P.o. *	P.o.
Seed dressing	-	+	+	+
Fertilization (kg/ha)	-	N-150, P-32, K-75	N-105, P-25, K-60	N-160, P-32, K-75
Herbicides	-	1 or 2x	1x	2 or 3x
Fungicides	-	2x	1 or 2x	2x
The growth regulator	-	+	+/-	+

Harrowing	2 or 3x	1x	1x	1x

*/ Z – potato, P.j. – spring wheat, P.o.- winter wheat, Rz. – winter rape, K.c.- red clover with grass, O+V - mix oats + vetch

Table S14. Calibration curve parameters for free phenolic acids reference standards.

No.	Phenolic acid	Calibration curve	R^2
1.	protocatechuic acid (PRO)	$y = -0.0254426\ x^2 + 1.46612\ x + 0.0137605$	0.997
2.	*p*-OH-benzoic acid (POCH)	$y = -0.0116753\ x^2 + 1.43904\ x + 0.164956$	0.997
3.	vanillic acid (VAN)	$y = 0.000116384\ x^2 + 0.194029\ x - 0.00311$	0.998
4.	caffeic acid (CAF)	$y = -0.0182712\ x^2 + 2.42109\ x + 0.436786$	0.995
5.	syringic acid (SYR)	$y = -0.0000546324\ x^2 + 0.259824\ x - 0.00264286$	0.986
6.	*p*-coumaric acid (PCO)	$y = -0.0165714\ x^2 + 2.05818\ x + 2.05818$	0.993
7.	ferulic acid (FER)	$y = -0.000407832\ x^2 + 0.380126\ x + 3.30005$	0.994
8.	sinapic acid (SIN)	$y = -0.00315979\ x^2 + 0.55236\ x - 0.0620381$	0.998
9.	salicylic acid (SAL)	$y = -0.0338427\ x^2 + 3.26378\ x + 0.82676$	0.998

Figure of Contents:

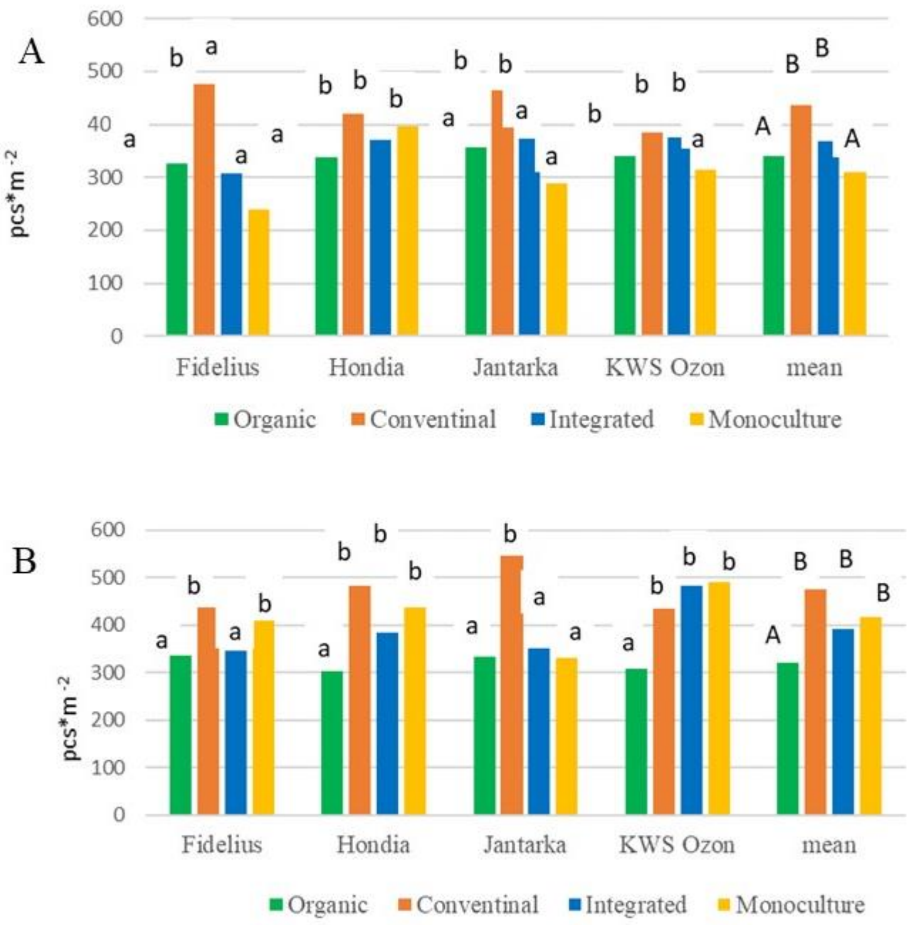

Figure S1. Effect of crop production systems on ears density of 4 winter wheat cultivars in 2-year study (A- 2017, B – 2018). *different letters in columns correspond to significant differences between means, according to Tukey's test at $p \leq 0.05$.

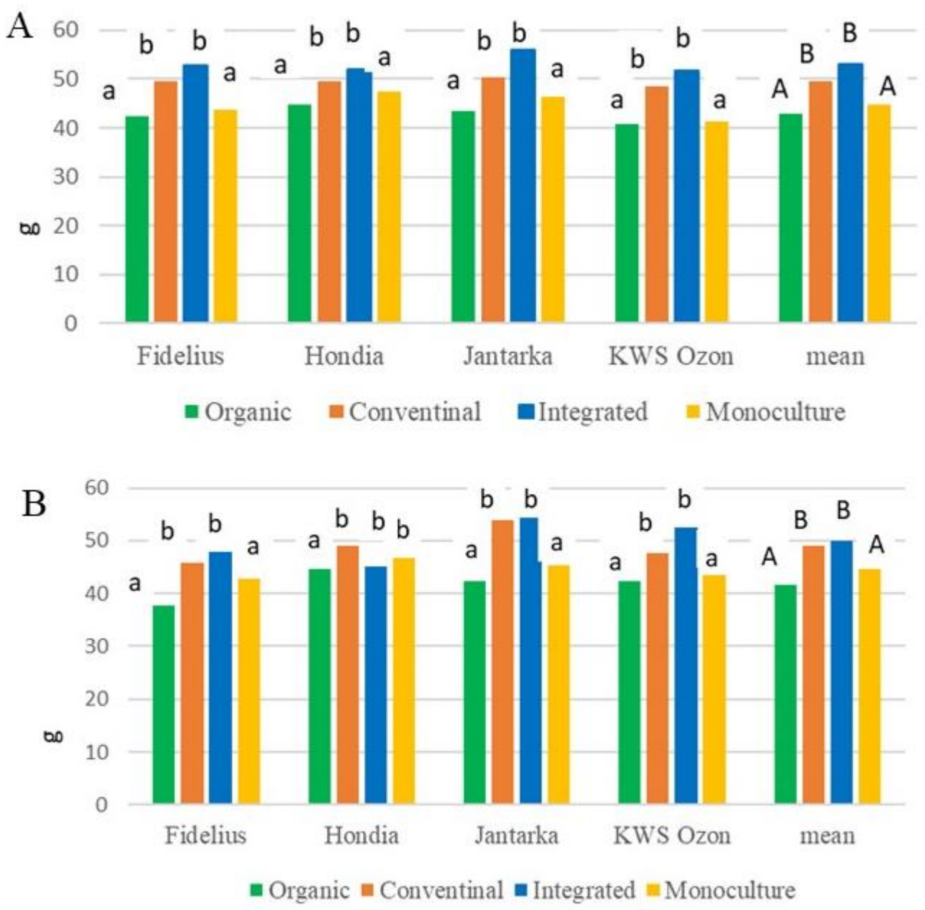

Figure S2. Effect of crop production systems on thousand grain mass of 4 winter wheat cultivars in a 2-year study (A- 2017, B – 2018). * different letters in columns correspond to significant differences between means, according to Tukey's test at $p \leq 0.05$.

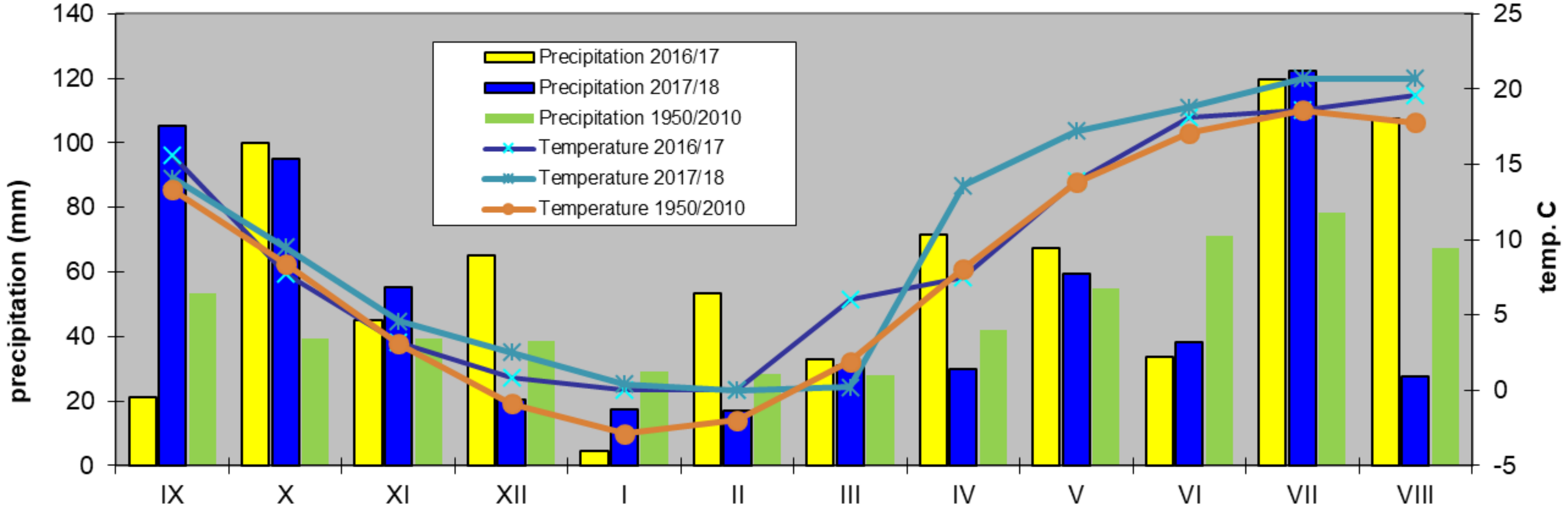

Figure S3. Monthly air temperature and monthly of precipitation in Osiny for the period of 2016–2018.
